# The effect of dietary carbohydrate and calorie restriction on weight and metabolic health in overweight/obese individuals: a multi-center randomized controlled trial

**DOI:** 10.1186/s12916-023-02869-9

**Published:** 2023-05-24

**Authors:** Jia Sun, Yuting Ruan, Ningning Xu, Peili Wu, Nie Lin, Kun Yuan, Shengli An, Pei Kang, Shu Li, Qiya Huang, Yingzhang, Yuzhong Li, Jialin Su, Wenjun Ma, Bo Chen, Xiuwei Zhang, Xiaoming Chen, Yongqian Liang, Zeyuan Lu, Guobao Deng, Zhen Zhang, Yuqin Wang, Weiheng Wen, Huijie Zhang, Hong Chen

**Affiliations:** 1 Department of Endocrinology and Metabolism, Zhujiang Hospital, Southern Medical University, No. 253, Industrial Avenue, Haizhu Square, Guangzhou, Guangdong China; 2 Department of Bio-Statistics, Southern Medical University, No.1023 Sha Tai Nan Lu, Baiyun Square, Guangzhou, Guangdong China; 3 Department of Endocrinology, Huizhou Municipal Center Hospital, No. 41, Eling North Road, Huizhou, Guangdong China; 4 Department of Endocrinology, Qing Yuan People’s Hospital, Qingyuan, Guangdong China; 5 Department of Endocrinology, The Third Affiliated Hospital of Guangzhou Medical University, No. 63, DuoBao Road, Liwan Square, Guangzhou, Guangdong China; 6 Department of Endocrinology, Dongguan Kanghua Hospital, 1000 Dongguan Avenue, Dongguan, Guangdong China; 7 Department of Endocrinology, He Xian Memorial Hospital, No. 2, Qinghe East Road, Panyu District, Guangzhou, Guangdong China; 8 Department of Nutrition, Guangdong Provincial People’s Hospital (Guangdong Academy of Medical Sciences), Southern Medical University, No. 106, Zhongshan Second Road, Yuexiu District, Guangzhou, Guangdong China; 9 Department of Endocrinology, Guangdong Second Provincial General Hospital, No.466, Xing Gang Middle Road, Haizhu District, Guangzhou, Guangdong China; 10 Department of Endocrinology, Dongguan People’s Hospital, Wandao Road (South), Xinguchong, Wanjiang District, Dongguan, Guangdong China; 11 Department of Endocrinology, Affiliated Hospital of Guangdong Medical University, No. 57, Renmin Avenue South, Xiashan District, Zhanjiang, Guangdong China; 12 Department of Endocrinology and Metabolism, Shunde Hospital of Southern Medical University, No. 1, Lunjiao Jiazi Road, Shunde District, Foshan, Guangdong China; 13 Department of Endocrinology, The Eighth Affiliated Hospital of Sun Yat-Sen University, No. 3025, Shennan Road, Shenzhen, Guangdong China; 14 Department of Endocrinology, Shaoguan First People’s Hospital, No. 3, Dongdi South Road, Zhenjiang District, Shaoguan, Guangdong China; 15 Pansonglou Health Management Center, Guangzhou First People’s Hospital, Guangzhou, China; 16 Department of Endocrinology and Metabolism, Nanfang Hospital, Southern Medical University, No. 1838 Guangzhou Dadao Bei, Baiyun Square, Guangzhou, Guangdong China

**Keywords:** Overweight/obese, Low-carbohydrate diet, Calorie-restricted diet, Weight loss, Metabolic risk factors

## Abstract

**Background:**

Both low‐carbohydrate (LC) and calorie-restricted (CR) diets have been shown to have metabolic benefits. However, the two regimens have yet to be thoroughly compared. We conducted a 12-week randomized trial to compare the effects of these diets separately and in combination on both weight loss and metabolic risk factors in overweight/obese individuals.

**Methods:**

A total of 302 participants were randomized to LC diet (*n* = 76), CR diet (*n* = 75), LC + CR diet (*n* = 76), or normal control (NC) diet (*n* = 75) using a computer-based random number generator. The primary outcome was the change in body mass index (BMI). The secondary outcomes included body weight, waist circumference, waist-to-hip ratio, body fat, and metabolic risk factors. All participants attended health education sessions during the trial.

**Results:**

A total of 298 participants were analyzed. BMI change over 12 weeks was − 0.6 (95% CI, − 0.8 to − 0.3) kg/m^2^ in NC, − 1.3 (95% CI, − 1.5 to − 1.1) kg/m^2^ in CR, − 2.3 (95% CI, − 2.6 to − 2.1) kg/m^2^ in LC, and − 2.9 (95% CI, − 3.2 to − 2.6) kg/m^2^ in LC + CR. LC + CR diet was more effective than LC or CR diet alone at reducing BMI (*P* = 0.001 and *P* < 0.001, respectively). Furthermore, compared with the CR diet, the LC + CR diet and LC diet further reduced body weight, waist circumference, and body fat. Serum triglycerides were significantly reduced in the LC + CR diet group compared with the LC or CR diet alone. Plasma glucose, homeostasis model assessment of insulin resistance, and cholesterol concentrations (total, LDL, and HDL) did not change significantly between the groups during the 12-week intervention.

**Conclusions:**

The reduction of carbohydrate intake without restricting caloric intake is more potent to achieve weight loss over 12 weeks when compared to a calorie-restricted diet in overweight/obese adults. The combination of restricting carbohydrate and total calorie intake may augment the beneficial effects of reducing BMI, body weight, and metabolic risk factors among overweight/obese individuals.

**Trial registration:**

The study was approved by the institutional review board of Zhujiang Hospital of Southern Medical University and registered at the China Clinical Trial Registration Center (registration number: ChiCTR1800015156).

**Supplementary Information:**

The online version contains supplementary material available at 10.1186/s12916-023-02869-9.

## Background

Obesity and its associated metabolic abnormalities have become a major public health challenge worldwide. From 1993 to 2015, obesity (BMI ≥ 27.5 m/kg^2^) increased from 4.2 to 15.7%, and abdominal obesity (≥ 90 cm for men and ≥ 80 cm for women) increased from 20.2 to 46.9% in China [1]. The overweight and obesity rate in adults has reached 34.3 and 16.4% according to the latest research, respectively [2]. It is estimated that China can expect a staggering 65.3% overweight/obesity by 2030 [3]. Obesity is characterized by excessive adipose tissue and is closely related to type 2 diabetes, hypertension, and cardiovascular disease [4]. Thus, a great deal of current research is focused on developing an optimal and effective treatment for obesity.

Dietary interventions have been proven to be an effective method for weight loss [4–7]. Traditionally, a calorie-restricted diet rich in fiber, with a percentage of total energy intake > 50% from carbohydrates and limited in fat, has been generally accepted and recommended by guidelines [8–10]. Calorie restriction improves metabolic health and glucose homeostasis [11, 12]. The beneficial effects of a calorie-restricted diet were believed to be the result of reduced caloric intake, although recent studies suggest that the reduction of specific macronutrients like carbohydrates may also provide viable treatment strategies in the management of obesity [13, 14]. Differences in the health benefits of a low-carbohydrate diet compared to a calorie-restricted diet are of considerable public interest. Several trials have found greater weight loss with a low-carbohydrate diet than with a low-calorie diet [15–18], while others reported no differences [19–22]. These findings complicate the interpretation of dietary intervention studies, as it is unclear which effects of weight loss result from reduced caloric intake, and which instead are attributable to the low carbohydrate.

In this study, we aimed to conduct a 12-week randomized controlled trial to compare the contribution of the weight loss effects of a calorie-restricted diet alone (low-calorie, high-to-moderate carbohydrate) with that of a low-carbohydrate diet alone (low-carbohydrate, without calorie restriction). We also evaluated whether the combination of dietary carbohydrates and calorie restriction could augment weight loss and improve the metabolic risk factors in overweight/obese individuals.

## Methods

### Study design and participants

This study was a multicenter, randomized, parallel-group clinical trial with participants allocated in a 1:1:1:1 ratio to normal control (NC) diet, low-carbohydrate (LC) diet, calorie-restricted (CR) diet, or LC + CR diet for 12 weeks. The randomization schedules were generated using a centrally controlled, computer-generated random number Internet-based system and concealed until an eligible participant was ready for enrollment.

All study participants were recruited from 13 hospitals in Guangdong Province, China, from April to July 2018. The trial protocol is available in Additional file 1 [4–6, 8–32]. The inclusion criteria were an age of 18–65 years and a body mass index (the weight in kilograms divided by the square of the height in meters) of at least 24 kg/m^2^. Individuals were excluded if they had a history of diabetes mellitus, cardiovascular and cerebrovascular disease, uncontrolled hypertension, gastrointestinal tract disorders, chronic hepatitis, gastrointestinal surgery, and allergies to proteins such as beans, wheat, and milk. In addition, participants were excluded if they took weight loss medications or anticipated a weight loss program.

The study was approved by the institutional review board of Zhujiang Hospital of Southern Medical University and registered at the China Clinical Trial Registration Center (https://www.chictr.org.cn), and the registration number is ChiCTR1800015156. All participants provided written informed consent before enrollment.

### Diet interventions

Participants in the NC group were designed to follow a non-restricted calorie diet with 55–65% of calories from carbohydrates, 30% from fat, and 20% from protein. The CR diet group restricted daily calories to 1200–1500 kcal/day with 55–65% of calories from carbohydrates, 30% from fat, and 20% from protein. Participants in the LC group were instructed to follow a non-restricted calorie diet with 26% of calories from carbohydrates, 50% from fat, and more than 24% from protein. The LC + CR group was instructed to restrict daily calories to 1200–1500 kcal/day with 26% of calories from carbohydrates, 50% from fat, and more than 24% from protein. Participants in the LC and LC + CR groups were provided with one nutritional bar (Nanda Fit Nutrition and Health Consulting Co., Ltd., Guangzhou, China) as the low-carbohydrate replacement at lunch and dinner and were instructed to consume low-carbohydrate grains, vegetables, fruits, and legumes. All the aliments were in accordance with dietary guidelines and previous studies for macronutrient intake [18, 23, 24, 28, 33]. Diets were structured to include specific foods. Additional file 2: Table S1 listed a quantitative food record that participants completed daily in different diet groups. All the participants met weekly for counseling sessions with dietitians who will provide dietary plans and advice over the 12-week period. The education session typically lasted 40 min and consisted of dietary advice, meal plans, and supportive counseling provided by registered dietitians for each participant.

All the participants were instructed to photograph the food they ate and write a daily dietary log to record macronutrients, nutrition labels, and detailed dietary recipes 3 days per week (including 2 working days and 1 weekend day). Using each participant’s log and their photographic identification of the food they ate, two researchers assessed participants’ nutrient intake on the basis of the nutrient content listed in the Chinese Food Composition Table [33]. Furthermore, participants were required to wear a Mi Band 2 Smart Bracelet (Xiaomi, Beijing, China) during the whole experimental process to record their regular physical activity. Participants were advised to do regular exercise 3 times a week with durations of 30 min. Adherence to the intervention program was defined according to the number of days that a participant met the requirements of the assigned diet. In addition, occurrences of any adverse event were collected throughout the study. Detailed recording of adverse events is available in Additional file 1.

### Outcomes and follow-up

The primary outcome was change in body mass index (BMI). The secondary outcomes included changes in body weight, body fat, waist circumference, waist-to-hip ratio (WHR), and metabolic risk factors. Body fat was quantified using IOI353 Body Composition Analyser (Jawon Medical, Gyeongsansi, South Korea). Blood samples were collected, after participants had fasted for over 8 h, to measure plasma glucose, fasting insulin, serum total cholesterol, serum triglyceride, high-density lipoprotein cholesterol (HDL-C), low-density lipoprotein cholesterol (LDL-C), uric acid, alanine aminotransferase (ALT), and aspartate aminotransferase (AST). Insulin resistance was evaluated by homeostasis model assessment-insulin resistance index (HOMA-IR), calculated as [plasma glucose (mmol/L) × serum insulin (mIU/L)]/22.5. All study outcomes were measured at baseline and weeks 4, 8, and 12.

### Statistical analysis

Based on data from previous studies [26, 27, 29, 30], this trial was designed to provide 85% power to detect a − 0.6 kg/m^2^ reduction in BMI [standard deviation (SD), − 0.6 kg/m^2^] between any 2 groups at 12 weeks at a significant level of 0.05 using a 2-tailed *t*-test. We also assumed an 80% follow-up rate.

All analyses were conducted in both the ITT and completer samples. A mixed-effects model was used to assess the effects of diet programs on the change in BMI, and an autoregressive correlation matrix was used to correct within-participant correlation for repeated measurements. In this model, participants were assumed to be random effects and intervention group, time and their 2-factor interactions, age, center, and sex were assumed to be estimable fixed effects that can be estimated. The PROC MIXED component of the SAS statistical software, version 9.4 (SAS Institute Inc.), was used to obtain point estimates and standard errors (SEs) of the treatment effects and to test for differences between treatments. Multiple imputations for missing data in the multivariable analyses were conducted using the Markov Chain Monte Carlo method. To determine the significant main effects and interactions between carbohydrates and calories, data were analyzed using factorial design ANOVA and two-way ANOVA using the general linear model procedures of SAS (Cary, NC). ANOVA results are presented in figure legends. When the interaction was not significant, the main effects were evaluated to assess the significance of the individual factors. Group differences in the study outcomes were evaluated using the general linear model for continuous variables and the chi-squared test for categorical variables. Data were presented as least-squares means with 95% confidence intervals (CIs) unless otherwise indicated. *P*-values < 0.05 was considered as indicating statistical significance.

## Results

### Baseline characteristic

**Fig. 1:**
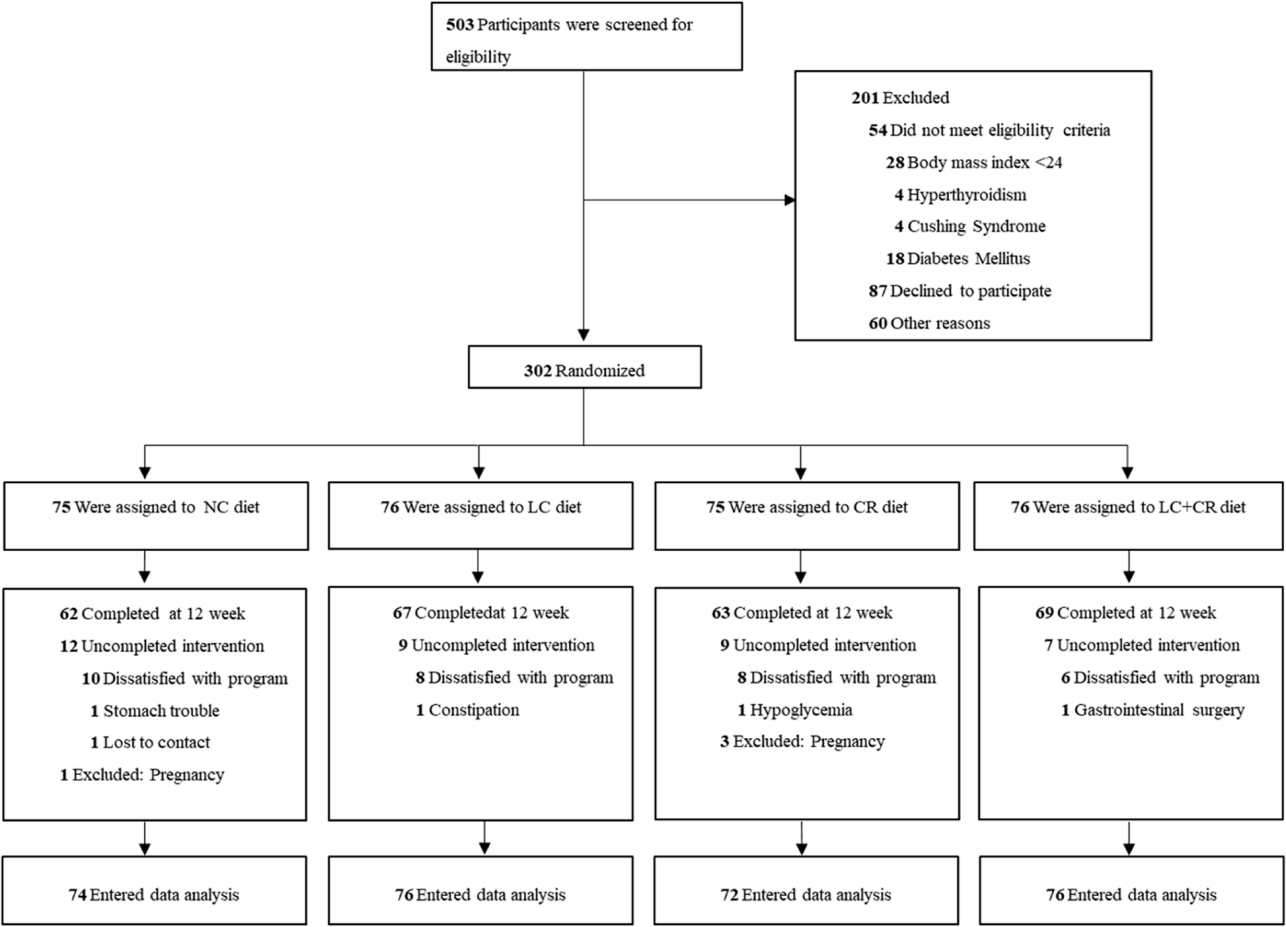
Flowchart of trial participants. NC, control; LC, low carbohydrate; CR, calorie restriction

The flow of participants is shown in Fig. 1. A total of 302 eligible overweight/obese participants (209 females, 93 males) were randomly assigned to the NC group (*n* = 75), LC group (*n* = 76), CR group (*n* = 75), and LC + CR group (*n* = 76). A total of 298 participants were included in the ITT analysis (Table 1). On average, the age of included individuals was 34.0 ± 7.3 years, body weight was 80.8 ± 15.1 kg, BMI was 30.3 ± 4.0 kg/m^2^, HOMA-IR was 3.5 ± 2.6, and FPG was 93.6 ± 9.2 mg/dL. Baseline characteristics were well balanced among the four groups. A total of 261 participants finally completed the 12-week follow-up visit were included in the completer analysis (Additional file 2: Table S2), with a dropout rate of 14.9% (NC group, 17.3%; LC group, 11.8%; CR group, 17.3%; LC + CR group 9.2%). Proportionally, more men (18.8%) than women (11.0%) dropped out, although these results were not statistically significant.

**Table 1:** Baseline characteristics of study participants^a^

Characteristic	NC diet (*n* = 74)	LC diet (*n* = 76)	CR diet (*n* = 72)	LC + CR diet (*n* = 76)
Female, no. (%)	48 (64.9)	53 (69.7)	52 (72.2)	52 (68.4)
Age, years	35.1 (8.2)	34.2 (7.8)	33.6 (6.4)	33.2 (7.0)
High school education, no. (%)	26 (35.1)	24 (31.6)	28 (38.9)	30 (39.5)
Current cigarette smoking, no. (%)	8 (10.8)	6 (7.9)	8 (11.1)	7 (9.2)
Current alcohol drinking, no. (%)	13 (17.6)	15 (19.7)	10 (13.9)	11 (14.5)
Total energy intake, kcal/day	1841.7 (436.4)	1792.9 (521.6)	1720.0 (449.4)	1783.3 (552.0)
Carbohydrate intake, %	50.3 (6.5)	48.1 (5.0)	50.6 (6.2)	51.7 (10.3)
Protein intake, %	21.3 (4.5)	21.6 (2.9)	18.2 (3.4)	18.2 (4.6)
Fat intake, %	28.3 (2.3)	30.3 (6.7)	30.4 (3.6)	31.0 (4.7)
Weight, kg	79.6 (14.0)	80.2 (14.1)	80.1 (13.1)	83.6 (18.9)
BMI, kg/m^2^	29.9 (3.9)	30.2 (3.8)	30.3 (3.4)	31.0 (4.7)
Waist circumference, cm	94.2 (10.3)	93.9 (10.0)	95.0 (9.3)	95.6 (13.7)
WHR	0.89 (0.06)	0.89 (0.05)	0.90 (0.06)	0.89 (0.07)
Heart rate, beats/min	77.0 (8.0)	78.7 (8.8)	76.8 (11.2)	76.8 (8.6)
Blood pressure, mmHg				
Systolic	118.4 (12.5)	114.9 (10.8)	115.0 (10.8)	115.5 (12.2)
Diastolic	75 (16.6)	76.7 (9.2)	79.54 (7.9)	77.9 (7.5)
Plasma glucose, mg/dL	94.3 (9.3)	92.0 (9.8)	93.9 (9.1)	93.8 (8.3)
HOMA-IR	3.6 (2.4)	3.5 (2.6)	3.2 (3.8)	3.7 (2.4)
Serum triglycerides, mg/dL	148.8 (83.1)	135.2 (82.5)	130.5 (94.0)	159.8 (123.3)
Serum total cholesterol, mg/dL	196.2 (36.3)	190.6 (38.4)	198.3 (38.2)	192.3 (36.8)
HDL, mg/dL	47.2 (11.3)	48.4 (13.2)	48.2 (9.6)	45.5 (9.8)
LDL, mg/dL	120.3 (31.4)	115.0 (33.5)	123.5 (31.6)	115.9 (26.8)
ALT, IU/L	27.5 (22.9)	25.2 (16.2)	27.5 (18.7)	29.1 (21.2)
AST, IU/L	21.7 (10.2)	20.1 (7.8)	20.7 (8.6)	20.9 (8.4)
Uric acid, mg/dl	6.7 (1.5)	6.5 (1.6)	6.7 (1.6)	6.8 (1.9)
Body fat, %	34.4 (4.5)	34.6 (5.1)	35.4 (4.3)	34.8 (4.1)
Visceral fat area, cm^2^	111.5 (40.3)	104.7 (34.5)	10.7 (31.9)	103.9 (45.3)
Body muscle rate, %	59.6 (8.2)	60.7 (8.2)	58.3 (11.5)	61.4 (7.2)

SI conversion factors: to convert glucose to millimoles per liter, multiply by 0.0555; to convert insulin to picomoles per liter, multiply by 6.945; to convert triglycerides to millimoles per liter, multiply by 0.0113; to convert total cholesterol, HDL-C, and LDL-C to millimoles per liter, multiply by 0.0259

*Abbreviations*: *BMI* Body mass index (calculated as the weight in kilograms divided by height in meters squared), *HOMA-IR* Homeostasis model assessment-insulin resistance index (calculated as [ plasma glucose (mmol/L) × serum insulin (mIU/L)]/22.5), *HDL* High-density lipoprotein, *LDL* Low-density lipoprotein, *ALT* Alanine aminotransferase, *AST* Aspartate aminotransferase, *WHR* Waist to hip ratio (calculated as the ratio of waist to hip circumference)

^a^Data are presented as mean (SD) unless otherwise indicated

### Dietary intake and adherence

The four groups reported similar physical activity during 12 weeks (Additional file 2: Table S3, *P* = 0.260). The dietary intake of energy and macronutrients during the 12-week intervention is shown in Table 2. There were no differences in calorie intake between the CR diet and LC + CR diet group (*P* = 0.211), whereas calorie intake was lower in the LC diet group than in the NC group (*P* = 0.006). In addition, there were no differences in carbohydrate intake between the LC diet group and the LC + CR diet group (*P* = 0.25) and between the NC group and the CR diet group (*P* = 0.445). Furthermore, the LC diet group had higher calorie intake than the CR diet group (*P* < 0.001), while the CR diet group had higher carbohydrate intake than the LC diet group (*P* < 0.001). The results for completer analyses were consistent with the ITT results (Additional file 2: Table S4).

**Table 2:** Energy and nutrition intake during follow-up

Characteristic	Changes (95% CIs)				*P* values				
	**NC diet (*n* = 74)**	**LC diet (*n* = 76)**	**CR diet (*n* = 72)**	**LC + CR diet (*n* = 76)**	**LC diet vs NC diet**	**LC + CR diet vs CR diet**	**CR diet vs NC diet**	**LC + CR diet vs LC diet**	**LC diet vs CR diet**
Energy intake, kcal									
Week 4	1654.3 (1555.8 to 1752.8)	1519.1 (1471.0 to 1567.1)	1217.2 (1142.9 to 1291.6)	1281.9 (1231.4 to 1332.4)	0.005	0.179	< 0.001	< 0.001	< 0.001
Week 8	1653.4 (1551.2 to 1755.7)	1510.3 (1463.8 to 1556.8)	1218.7 (1141.6 to 1295.8)	1283.0 (1235.2 to 1330.9)	0.004	0.187	< 0.001	< 0.001	< 0.001
Week 12	1639.0 (1535.9 to 1742.1)	1503.6 (1459 to 1548.2)	1217.1 (1140.0 to 1294.2)	1278.0 (1229.9 to 1326.2)	0.006	0.211	< 0.001	< 0.001	< 0.001
Carbohydrate intake, g									
Week 4	203.3 (187.1 to 219.5)	63.1 (60.6 to 65.6)	145.8 (133.9 to 157.8)	57.6 (53.3 to 61.9)	< 0.001	< 0.001	< 0.001	0.4	< 0.001
Week 8	199.8 (182.8 to 216.9)	63.7 (60.8 to 66.6)	145.6 (133.1 to 158)	58.5 (54.1 to 62.9)	< 0.001	< 0.001	< 0.001	0.475	< 0.001
Week 12	202.8 (185.6 to 220)	62.8 (60.4 to 65.2)	146.7 (134.1 to 159.2)	57.9 (53.7 to 62.1)	< 0.001	< 0.001	< 0.001	0.448	< 0.001
Carbohydrate intake, %									
Week 4	48.8 (46.9 to 50.8)	16.7 (16.1 to 17.3)	47.6 (45.7 to 49.6)	17.9 (17.1 to 18.6)	< 0.001	< 0.001	0.235	0.212	< 0.001
Week 8	48.6 (46.7 to 50.6)	16.7 (16.2 to 17.2)	47.8 (45.8 to 49.9)	17.9 (17.2 to 18.7)	< 0.001	< 0.001	0.436	0.179	< 0.001
Week 12	48.3 (46.6 to 50.1)	17.1 (16.1 to 18.1)	47.5 (45.5 to 49.6)	18.2 (17.4 to 19.0)	< 0.001	< 0.001	0.445	0.25	< 0.001
Protein intake, g									
Week 4	36.8 (33.6 to 40)	55.4 (53 to 57.8)	27.1 (24.6 to 29.7)	47.4 (45 to 49.8)	< 0.001	< 0.001	< 0.001	< 0.001	< 0.001
Week 8	35.9 (33 to 38.8)	55.8 (53.3 to 58.3)	27.7 (24.9 to 30.5)	47 (44.7 to 49.2)	< 0.001	< 0.001	< 0.001	< 0.001	< 0.001
Week 12	36.4 (33.5 to 39.3)	55.6 (53.1 to 58.1)	27.7 (24.8 to 30.6)	47.3 (45.1 to 49.5)	< 0.001	< 0.001	< 0.001	< 0.001	< 0.001
Protein intake, %									
Week 4	20.0 (18.8 to 21.2)	32.9 (31.9 to 33.8)	20.2 (18.4 to 21.9)	33.1 (32.4 to 33.8)	< 0.001	< 0.001	0.835	0.746	< 0.001
Week 8	19.8 (18.8 to 20.9)	33.1 (32.2 to 34.0)	20.6 (18.5 to 22.6)	33.1 (32.4 to 33.8)	< 0.001	< 0.001	0.423	0.981	< 0.001
Week 12	19.7 (18.7 to 20.7)	33.5 (32.2 to 34.7)	20.6 (18.7 to 22.5)	33.0 (32.3 to 33.7)	< 0.001	< 0.001	0.331	0.569	< 0.001
Fat intake, g									
Week 4	59.9 (56.0 to 63.9)	84.0 (80.1 to 87.9)	48.3 (45.6 to 50.9)	67.6 (65.4 to 69.9)	< 0.001	< 0.001	< 0.001	< 0.001	< 0.001
Week 8	60.1 (56.4 to 63.8)	82.8 (79.6 to 86.0)	48.1 (45.5 to 50.6)	67.7 (65.5 to 69.9)	< 0.001	< 0.001	< 0.001	< 0.001	< 0.001
Week 12	59.8 (56.3 to 63.3)	82.8 (80.1 to 85.6)	48.2 (45.7 to 50.8)	67.3 (65.1 to 69.5)	< 0.001	< 0.001	< 0.001	< 0.001	< 0.001
Fat intake, %									
Week 4	32.9 (31.3 to 34.4)	49.7 (48.6 to 50.7)	36.2 (34.6 to 37.7)	47.7 (46.9 to 48.5)	< 0.001	< 0.001	< 0.001	0.019	< 0.001
Week 8	33.0 (31.5 to 34.6)	49.3 (48.4 to 50.3)	36.0 (34.6 to 37.4)	47.7 (46.9 to 48.4)	< 0.001	< 0.001	< 0.001	0.031	< 0.001
Week 12	33.2 (31.9 to 34.6)	49.7 (48.5 to 50.9)	36.2 (34.8 to 37.6)	47.5 (46.8 to 48.2)	< 0.001	< 0.001	< 0.001	0.006	< 0.001

During the 12-week intervention, the mean (± SD) percentage of the days that participants adhered to both the prescribed calories and carbohydrates was 84.9 ± 32.3% in the NC group, 90.3 ± 21.9% in the LC group, 84.6 ± 26.2% in the CR group, and 87.2 ± 24.2% in the LC + CR group. No significant difference was observed between the groups over 12 weeks (*P* = 0.626, Additional file 2: Fig. S1 and Table S5).

### Weight loss and body composition

**Fig. 2:**
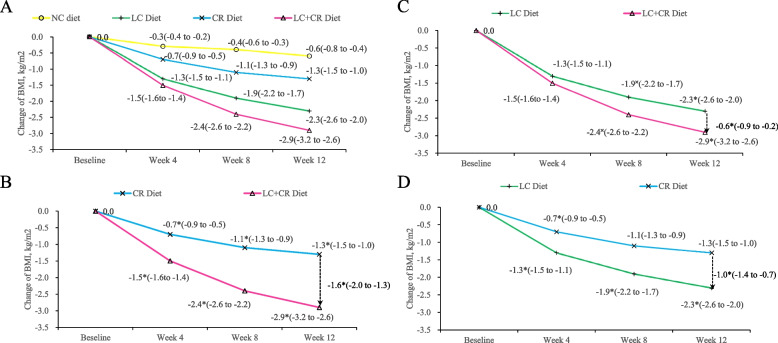
Effects of different dietary intake on BMI. **A** Changes in BMI over 12 weeks in 4 groups. **B** Changes and net change in BMI over 12 weeks in the LC diet groups. The net change in BMI in the LC diet group was −0.6 (−0.9, −0.2) kg/m^2^ compared with the LC+CR diet group (*P* < 0.001). **C** Changes and net change in BMI over 12 weeks in the CR diet groups. The net change in BMI in the CR diet group was − 1.6 (−2.0, −1.3) kg/m^2^ compared with the LC+CR diet group (*P* < 0.001). **D** Changes and net change in BMI over 12 weeks in the LC diet group and CR diet group. The net change in BMI in the LC diet group was −1.0 (−1.4, −0.7) kg/m^2^ compared with the CR diet group (*P* < 0.001). Total numbers for each diet group are as follows: NC diet, *n*=74; LC diet, *n*=76; CR diet, *n*=72; LC + CR diet, *n*=76. NC, control; LC, low carbohydrate; CR, calorie restriction. Numbers in parentheses are 95% CIs. ^×^*P* < 0.05, *P < 0.001

BMI change over 12 weeks was − 0.6 (− 0.8, − 0.3) kg/m^2^ in NC diet, − 1.3 (− 1.5, − 1.1) kg/m^2^ in CR diet, − 2.3 (− 2.6, − 2.1)kg/m^2^ in LC diet, and − 2.9 (− 3.2, − 2.6) kg/m^2^ in LC + CR diet (*P* < 0.001 for all, Table 3). Despite CR and LC + CR diets reducing similar calorie intake, there were clear differences between the effect of diets. The BMI reductions were significantly greater with the LC + CR than the CR diet when compared with the NC group (*P* < 0.001). When comparing the weight loss effect between calorie restriction and low carbohydrate, we found that BMI reduction is stronger in LC diet than in CR diet. The net change in BMI in the LC diet group was − 1.0 (− 1.4, − 0.7) kg/m^2^ (*P* < 0.001) compared with the CR diet group. The LC + CR diet group lost 55.1% more BMI than the CR diet group (− 2.3 [− 2.7, − 2.0] kg/m^2^ vs − 0.7 [− 1.1, − 0.3] kg/m^2^, respectively, *P* < 0.001) and lost 21.5% more BMI than the LC diet group (− 2.3 [− 2.7, − 2.0] kg/m^2^ vs − 1.7 [− 2.1, − 1.4] kg/m^2^, respectively, *P* = 0.001, Fig. 2).

**Table 3:** Effects of dietary intake on weight loss and body fat after the 12-week intervention

Outcomes	Changes (95% CIs)				*P* Values				
	**NC diet (*n* = 74)**	**LC diet (*n* = 76)**	**CR diet (*n* = 72)**	**LC + CR diet (*n* = 76)**	**LC diet vs NC diet**	**LC diet vs CR diet**	**LC + CR diet vs LC diet**	**LC + CR diet vs CR diet**	**Group differences**
BMI, kg/m^2^	− 0.6 (− 0.8 to − 0.3)	− 2.3 (− 2.6 to − 2.1)	− 1.3 (− 1.5 to − 1.0)	− 2.9 (− 3.2 to − 2.6)	< 0.001	< 0.001	0.001	< 0.001	< 0.001
Weight, kg	− 1.5 (− 2.2 to − 0.8)	− 5.9 (− 6.6 to − 5.2)	− 3.3 (− 4.0 to − 2.6)	− 7.8 (− 8.5 to − 7.2)	< 0.001	< 0.001	< 0.001	< 0.001	< 0.001
Waist circumference, cm	− 1.7 (− 2.4 to − 0.9)	− 5.5 (− 6.2 to − 4.7)	− 3.3 (− 4.0 to − 2.5)	− 6.9 (− 7.6 to − 6.1)	< 0.001	< 0.001	0.009	< 0.001	< 0.001
WHR ratio	− 0.01 (− 0.01 to 0.00)	− 0.03 (− 0.03 to − 0.02)	− 0.02 (− 0.03 to − 0.01)	− 0.03 (− 0.03 to − 0.02)	< 0.001	0.155	0.358	0.020	< 0.001
Body fat, %	− 0.6 (− 1.1 to − 0.1)	− 2.5 (− 3.0 to − 2.0)	− 1.5 (− 2.0 to − 1.0)	− 3.0 (− 3.5 to − 2.6)	< 0.001	0.004	0.110	< 0.001	< 0.001

*Abbreviations*: *BMI* Body mass index (calculated as the weight in kilograms divided by height in meters squared), *WHR* Waist to hip ratio (calculated as the ratio of waist to hip circumference)

Among participants who began the low-carbohydrate diet, 77.6% achieved the target of ≥ 5% weight loss and 17.1% achieved the target of ≥ 10% weight loss at 12 weeks. Similarly, the LC + CR diet group showered a large reduction in body weight than either the LC diet group (− 2.0 [− 2.9, − 1.0] kg, *P* < 0.001) or the CR diet group (− 4.5[− 5.5, − 3.6] kg, *P* < 0.001). But there was still a significant difference between the changes in the LC and CR groups, with the net change in body weight in the LC diet group being − 2.6 (− 3.5, − 1.6) kg compared with the CR diet group at 12 weeks (*P* < 0.001, Table 3).

We examined the contribution of carbohydrate and calorie restriction on body composition. We found all three restricted diets reduced waist circumference significantly after 12 weeks. Reduction was significantly greater with LC + CR than LC diet (− 1.4 [-2.4, − 0.4] cm, *P* = 0.009) or CR diet (− 3.6 [− 4.7, − 2.6] cm, *P* < 0.001). Similarly, waist circumference was reduced more in the LC diet compared with the CR diet (− 2.2 [− 3.3, − 1.2] cm, *P* < 0.001). Besides, the LC + CR diet group showed significant larger reductions in WHR (− 0.02 [− 0.03, − 0.01], *P* = 0.020] and body fat percentage (− 2.4 [− 3.1, − 1.8] %, *P* < 0.001) than the CR diet group (Table 3).

Completer analyses also showed that the LC diet group lost more weight than people in the CR diet group (− 6.1 [− 10.8, − 1.3] kg vs − 3.1 [− 8.0, 1.8] kg, *P* < 0.001), although energy intake throughout the intervention was significantly lower with CR. Weight reduction was largest among LC + CR diet when compared with CR or LC diet alone (Additional file 2: Table S6).

### Metabolic risk factors

Fasting glucose, HOMA-IR, total cholesterol, HDL-cholesterol, and LDL cholesterol decreased to the same extent between the three dietary interventions. We observed that fasting triacylglycerol was significantly improved only in the LC + CR diet intervention (*P* < 0.001) and not in LC diet (*P* = 0.19) or CR diet (*P* = 0.704). Besides, uric acid was significantly reduced in three different diet group (Table 4, *P* < 0.001 for all). The results for completer analyses were consistent with the ITT results (Additional file 2: Table S7).

**Table 4:** Effects of dietary intake on cardiovascular risk factors during the 12-week intervention^a^

Outcome	Between-group differences from baseline to week 12						*P* value of group differences
	**LC diet vs NC diet**		**CR diet vs NC diet**		**LC + CR diet vs NC diet**		
	**Changes (95% CIs)**	***P* value**	**Changes (95% CIs)**	***P* value**	**Changes (95% CIs)**	***P* value**	
Plasma glucose, mg/dL							
Week 4	− 0.7 (− 3.4 to 2.1)	0.635	− 2.0 (− 4.8 to 0.9)	0.172	− 3.7 (− 6.5 to − 0.9)	0.01	0.049
Week 8	− 0.5 (− 3.2 to 2.2)	0.732	− 1.4 (− 4.1 to 1.4)	0.327	− 3.0 (− 5.7 to − 0.3)	0.028	0.128
Week 12	− 0.7 (− 3.8 to 2.5)	0.679	− 2.5 (− 5.8 to 0.7)	0.119	− 0.6 (− 3.8 to 2.5)	0.702	0.436
HOMA-IR							
Week 4	− 0.9 (− 1.8 to − 0.1)	0.03	− 0.4 (− 1.3 to 0.4)	0.321	− 1.3 (− 2.1 to − 0.4)	0.003	0.016
Week 8	− 0.5 (− 1.4 to 0.3)	0.219	0.4 (− 0.5 to 1.3)	0.354	− 0.8 (− 1.7 to 0.1)	0.065	0.028
Week 12	− 0.4 (− 1.3 to 0.4)	0.277	0.2 (− 0.6 to 1.0)	0.587	− 0.6 (− 1.4 to 0.2)	0.174	0.187
Serum triglycerides, mg/dL							
Week 4	10.3 (− 20.8 to 41.5)	0.515	19.8 (− 11.8 to 51.4)	0.219	− 39.4 (− 70.5 to − 8.2)	0.013	0.001
Week 8	− 8.1 (− 32.6 to 16.5)	0.519	− 1.2 (− 26.1 to 23.7)	0.923	− 44.6 (− 69.2 to − 20.1)	< 0.001	0.001
Week 12	− 21.9 (− 54.6 to 10.9)	0.19	6.4 (− 39.6 to 26.8)	0.704	− 56.3 (− 89.0 to − 23.5)	< 0.001	0.001
Serum total cholesterol, mg/dL							
Week 4	− 3.6 (− 12.1 to 4.9)	0.406	− 1.1 (− 9.7 to 7.4)	0.794	− 3.8 (− 12.3 to 4.6)	0.375	0.77
Week 8	− 5.3 (− 63.3 to 52.8)	0.858	49.7 (− 9.2 to 108.5)	0.098	12.0 (− 46.0 to 70.1)	0.684	0.25
Week 12	10.2 (1.1 to 19.4)	0.029	− 0.4 (− 9.7 to 8.9)	0.937	− 0.3 (− 9.5 to 8.9)	0.948	0.056
HDL-C, mg/dL							
Week 4	− 0.9 (− 3.6 to 1.7)	0.49	− 0.9 (− 3.6 to 1.8)	0.524	0.5 (− 2.2 to 3.1)	0.731	0.674
Week 8	0.2 (− 2.6 to 3.0)	0.891	0.1 (− 2.8 to 2.9)	0.969	1.1 (− 1.7 to 4.0)	0.426	0.839
Week 12	3.4 (0.0 to 6.8)	0.048	1.6 (− 1.9 to 5.0)	0.373	2.3 (− 1.1 to 5.7)	0.185	0.249
LDL-C, mg/dL							
Week 4	− 5.5 (− 12.6 to 1.5)	0.124	− 6.6 (− 13.7 to 0.6)	0.073	− 1.4 (− 8.5 to 5.6)	0.693	0.207
Week 8	− 1.1 (− 8.6 to 6.5)	0.779	− 5.1 (− 12.8 to 2.5)	0.186	2.1 (− 5.4 to 9.7)	0.58	0.294
Week 12	5.4 (− 2 to 12.9)	0.151	− 5.3 (− 12.9 to 2.2)	0.165	− 0.3 (− 7.7 to − 7.2)	0.943	0.047
ALT, IU/L							
Week 4	− 3.8 (− 7.8 to 0.1)	0.055	0.1 (− 3.9 to 4.1)	0.952	− 2.3 (− 6.2 to 1.7)	0.257	0.142
Week 8	− 5.0 (− 9.2 to − 0.7)	0.022	− 3.2 (− 7.4 to 1.1)	0.149	− 7.1 (− 11.3 to − 2.8)	0.001	0.01
Week 12	− 6.5 (− 11.6 to − 1.4)	0.013	− 2.2 (− 7.4 to 3.0)	0.408	− 7.0 (− 12.1 to − 1.9)	0.007	0.018
AST, IU/L							
Week 4	− 1.1 (− 3.7 to 1.5)	0.394	− 0.3 (− 2.9 to 2.4)	0.851	0.3 (− 2.3 to 2.8)	0.847	0.74
Week 8	− 0.2 (− 2.8 to 2.4)	0.885	− 0.2 (− 2.9 to 2.5)	0.897	− 0.4 (− 3.1 to − 2.2)	0.743	0.991
Week 12	− 3.7 (− 6.7 to − 0.7)	0.017	− 1.2 (− 4.2 to 1.9)	0.453	− 2.7 (− 5.7 to 0.3)	0.081	0.083
Uric acid, mg/dL							
Week 4	− 0.3 (− 0.7 to 0.1)	0.116	− 0.3 (− 0.7 to 0.1)	0.199	− 0.4 (− 0.8 to 0)	0.076	0.821
Week 8	− 0.8 (− 1.2 to − 0.4)	< 0.001	− 0.5 (− 0.9 to − 0.1)	0.02	− 0.6 (− 1.0 to − 0.2)	0.004	0.005
Week 12	− 0.8 (− 1.2 to − 0.4)	< 0.001	− 0.6 (− 1.0 to − 0.2)	0.003	− 0.8 (− 1.2 to − 0.4)	< .001	0.002
Visceral fat area, cm^2^							
Week 4	− 5.1 (− 8.6 to 1.6)	0.005	− 3.9 (− 7.5 to 0.4)	0.031	− 8.5 (− 12.0 to 5.0)	< 0.001	< 0.001
Week 8	− 12.6 (− 16.3 to − 8.8)	< 0.001	− 7.4 (− 11.2 to − 3.6)	< 0.001	− 17.8 (− 21.5 to − 14.0)	< 0.001	< 0.001
Week 12	− 14.8 (− 19.3 to − 10.3)	< 0.001	− 8.4 (− 13.0 to − 3.8)	< 0.001	− 21.7 (− 26.2 to − 17.2)	< 0.001	< 0.001
Body muscle rate, %							
Week 4	1.6 (− 1.0 to 4.3)	0.223	− 1.5 (− 4.3 to 1.1)	0.249	1.9 (− 0 8 to 4.5)	0.165	0.040
Week 8	1.6 (− 1.7 to 4.8)	0.335	− 1.3 (− 4.6 to 2.0)	0.437	3.5 (0.3 to 6.8)	0.032	0.023
Week 12	3.8 (1.8 to 5.7)	< 0.001	0.7 (− 1.3 to 2.80)	0.475	3.3 (1.3 to 5.2)	0.001	< 0.001

*Abbreviations*: *BMI* Body mass index (calculated as the weight in kilograms divided by height in meters squared), *HOMA-IR* Homeostasis model assessment-insulin resistance index (calculated as [ plasma glucose (mmol/L) × serum insulin (mIU/L)]/22.5), *HDL-C* High-density lipoprotein cholesterol, *LDL-C* Low-density lipoprotein cholesterol, *ALT* Alanine aminotransferase, *AST* Aspartate aminotransferase, *WHR* Waist to hip ratio (calculated as the ratio of waist to hip circumference)

^a^Data are presented as mean (95%CIs)

### Adverse events

No deaths or serious adverse events were reported throughout the study. One participant in the CR diet group reported asymptomatic hypoglycemia and one in the LC + CR diet group reported gastrointestinal surgery because of small polyps.

## Discussion

In this multicenter study, we test the hypothesis that a reduction of carbohydrate intake without restricting caloric intake was more effective on weight loss when compared to a calorie-restricted diet in overweight/obese adults. Our findings align with some previous studies. Samaha et al. [17] studied 132 participants with severe obesity over 6 months and reported that those on a low-carbohydrate diet lost more weight than those on a calorie- and fat-restricted diet. Bazzano et al. [18] and Gardner et al. [15] reported that a low-carbohydrate diet was associated with significantly greater weight loss and reduction in BMI than a low-calorie diet over 12 months of intervention. Furthermore, Shai et al. [16] studied 322 participants with moderate obesity and reported that over a 24-month period, a low-carbohydrate diet resulted in greater weight loss than a low-calorie (low-fat) diets. However, these studies have examined the weight loss effects of a low-carbohydrate diet with a low-calorie and low-fat diet, but most of these trials did not compare the weight loss effects of a calorie-restricted diet alone with that of a low-carbohydrate diet without calorie restriction. Therefore, we innovatively designed the randomized clinical trial to compare an isolated calorie-restricted diet, an isolated low-carbohydrate diet, and the combination of carbohydrate and calorie restriction diet to dissect the effect of calories and carbohydrates on weight loss and other metabolic outcomes.

Our results indicated that a low-carbohydrate diet alone without calorie restriction is sufficient to improve body weight, waist circumference, and body fat when compared with a calorie-restricted diet after a 12-week intervention. Evidence indicates that weight loss via lifestyle modification is largely mediated by energy intake rather than energy expenditure [34]. Although energy deficit is comparable in the three interventional groups, participants in the low carbohydrate group lost more weight in this study. One possibility is that low-carbohydrate diets may have a more favorable effect on energy expenditure than low-fat diets and high-carbohydrate diets [35, 36]. Previous studies indicated that lowering dietary carbohydrates with the same energy intake increased rest energy expenditure during weight loss maintenance, with a linear trend of 52 kcal/day for every 10% decrease in the contribution of carbohydrates to total energy intake [35]. In addition, we observed two acylcarnitine metabolites increased significantly in LC diet than in CR diet after 12 weeks of intervention (Additional file 2: Fig. S2). Acylcarnitines are intermediaries of FA and amino acid catabolism. Adipose tissue lipolysis promotes hepatic acylcarnitine production. The elevating level of acylcarnitine metabolites indicated more fat mobilization and energy expenditure in LC diet. Another explanation is that we observed nearly 50% of participants produced ketones in the LC group and LC + CR group by using daily urine ketone test strips (data not shown). It is likely that the weight loss relates, at least in part, to the known anorexigenic effects of ketone bodies [37]. However, the underlying mechanisms that may account for differences in weight loss by diet still need to be validated in larger sample sizes and future clinical trials. A new perspective on the relationship between diet and weight loss has emerged in recent years whereby the beneficial effects of dietary regimens may not be due to calories alone but rather to a combination of total caloric intake, the ratio of micronutrition, and their interaction. In our study, no interaction between carbohydrate and calorie level for weight loss was observed, and most metabolic factors were not statistically significant (Additional file 2: Table S8); thus, the main effects were evaluated to assess the significance of the individual factors. Our data presented here suggest that the combination of carbohydrate and calorie restriction achieved more weight loss than a low-carbohydrate or calorie-restricted diet alone. Additionally, the restriction of calories without reducing carbohydrate intake had a muted impact on weight loss and metabolic risk factors when compared with LC and LC + CR diets. Taken together, our results indicated that the reduction of total energy intake from carbohydrates to 26% below was necessary for a calorie-restricted diet to improve body weight, which has important implications for clinical practice.

Several clinical trials assessed the effects of low-carbohydrate diet and calorie-restricted diet on waist circumference and body fat among obese patients. Bazzano et al. [18] found that a low-carbohydrate diet had more favorable changes in waist circumference compared with a low-calorie diet, while there were no differences in body fat over 3 months of intervention. In contrast, Dansinger et al. [22] tested the effectiveness of four popular diets on weight loss in a 1-year randomized trial among 160 obese adults and reported no significant differences in waist circumference between low-carbohydrate diet and calorie-restricted diet. Gardner et al. [15] and colleagues also reported that a low-carbohydrate diet achieved a greater reduction in body fat than a calorie-restricted diet over 6 months. Our findings indicated that a low-carbohydrate diet significantly reduced more waist circumference and body fat than a calorie-restricted diet. Furthermore, the low-carbohydrate calorie-restricted diet reduced more waist circumference and body fat than the calorie-restricted diet alone. Our study suggests that the combination of carbohydrate and calorie restriction could be recommended for rapid improvement in fat deposition in overweight/obese individuals.

In this trial, only a low-carbohydrate calorie-restricted diet significantly reduced serum triglycerides during the 12-week intervention while other diets had no effect on lipids. In contrast, Samaha and colleagues [17] reported that a low-carbohydrate diet had a greater decrease in serum triglycerides than a calorie- and fat-restricted (low-fat) diet in 132 severely obese subjects. However, the dietary intervention did not significantly improve the level of HDL-C, LDL-C, total cholesterol, and glucose in our study. It is notable that the low-carbohydrate diets showed elevated total and/or LDL cholesterol, which may be resulted from their intake of saturated fatty acids and animal protein was not strictly controlled.

This study has some limitations. Firstly, the results presented in this analysis provide data only on short-term changes, maintaining weight loss and accompanying improvements is challenging. Whether differences between diets persist in the long term still needs to study. Secondly, the provision of nutritional bar might affect adherence, which might have biased the results. However, adherence to diet intervention did not differ between these groups. Thirdly, physical activity was not strictly controlled in this study because we aimed to examine the isolated effect of low-carbohydrate and calorie-restricted diets on weight loss. Finally, objective biomarkers of energy and macronutrient intake are lacking. Stronger weight loss with LC diet than CR diet despite higher calorie intake with LC is surprising and requires follow-up in controlled biomarker studies. Energy expenditure measured by the double-labeled water method may help to explain the differences among patients in weight loss in response to the diet interventions.

## Conclusions

In this study, we dissect the effects of calories and carbohydrates and highlight the important of carbohydrate restriction, and not solely reduced caloric intake is more important to achieve weight loss over a 12-week period. The combination of restricting carbohydrate and total calorie intake may augment the beneficial effects of reducing BMI, body weight, and metabolic risk factors among overweight/obese individuals.

## Supplementary Information

Additional file 1. The trial protocol.

Additional file 2: Fig. S1. Adherence to the prescribed diets over 12 weeks. Fig. S2. Metabolites changes of LC diet and CR diet at baseline and 12 weeks. Table S1. Food profile of diet interventions. Table S2. Baseline characteristics of study participants included in completer analysis. Table S3. Daily physical activityat baseline and during follow-up. Table S4. Energy and nutrition intake during follow-up of completer participants. Table S5. Adherence during the 12-week intervention. Table S6. Effects of dietary intake on weight loss and body fat after  -week intervention of completer participants. Table S7. Effects of dietary intake on cardiovascular risk factors during 12-week intervention of completer participants. Table S8. Tests of between subject-effect.

## Data Availability

Please contact the corresponding authors to discuss the availability of the datasets used and/or analyzed during the current study.

## Abbreviations

ALT: Alanine aminotransferase
ANOVA: Analysis of variance
AST: Aspartate aminotransferase
BMI: Body mass index
CIs: Confidence intervals
CR: Calorie-restricted
HDL: High-density lipoprotein
HOMA-IR: Homeostasis Model Assessment-Insulin Resistance Index
LC: Low‐carbohydrate
LDL: Lipoprotein cholesterol
SD: Standard deviation
SEs: Standard errors
WHR: Waist-to-hip ratio
