# Supplementary material for: The effect of dietary carbohydrate and calorie restriction on weight and metabolic health in overweight/obese individuals: a multi-center randomized controlled trial
Source: BMC Med. 2023 May 24;21:192. doi: 10.1186/s12916-023-02869-9 (PMC10210464; doi:10.1186/s12916-023-02869-9)
Supplement: Supplementary file 1 — Additional file 1. The trial protocol. [file 12916_2023_2869_MOESM1_ESM.pdf]

The effect of dietary carbohydrate and calorie restriction on weight and metabolic health in overweight/obese individuals: A multi-center randomized controlled trial

(A Randomized, Multicenter, Controlled Trial)

STUDY PROTOCOL

Zhujiang Hospital, Southern Medical University

Nanfang Hospital, Southern Medical University

School of Public Health and Tropical Medicine, Southern Medical University

ABBREVIATIONS

BMI: Body mass index

CRF: Case report form

ECG: Electrocardiogram

BP: Blood pressure

DBP: Systolic blood pressure

SBP: Diastolic blood pressure

HDL-C: High-density lipoprotein cholesterol

LDL-C: Low-density lipoprotein cholesterol

HOMA-IR: Homeostasis model assessment-insulin resistance

TC: Total cholesterol

TG: Triglyceride

WHR: Waist to hip ratio

LC: Low-carbohydrate

CR: Calorie-restricted

NC: Normal control

1. Background and Significance

Obesity and its associated metabolic abnormalities have become a major public health challenge worldwide. It affected 12.0% of adults around the world[23] and 14.0% in China[24]. Obesity is characterized by excessive adipose tissue and is closely related to type 2 diabetes, hypertension and cardiovascular disease[4]. Thus, seeking an optimal effective treatment for obesity is currently a hot topic among researchers.

Dietary interventions have been proven to be an effective treatments targeting weight loss[4-6]. The conventional dietary approach following a high/moderate-carbohydrate, low-fat, energy-deficit diet to achieve weight loss is generally accepted and recommended by guidelines[8-10]. Calorie-restricted diet has been recognized as the main component of weight loss intervention[11-12]. In recent decades, low-carbohydrate diet has been compared with conventional energy-restricted diets, and become a popular strategy for weight loss and weight maintains[13-14]. The differences in health benefits between a low-carbohydrate diet and a calorie-restricted diet are of considerable public interest. Several clinical trials assessed the weight loss effects between low-carbohydrate diet and calorie-restricted diet. Several clinical trials assessed the weight loss effects between low-carbohydrate diet and calorie-restricted diet. Dansinger[20] and colleagues found no statistically significant differences between low-carbohydrate diet and calorie-restricted diet during the 12 months intervention. Foster[21, 22] and Stern[19] also reported that there were no significant differences in weight loss between low-carbohydrate diet and calorie-restricted diet at 12 months. In contrast, Samaha[16] found that subjects on a low-carbohydrate diet lost more weight than those on a calorie and fat-restricted diet in 132 severely obese participants during the 6-month study period. Bazzano[15] and Gardner[18] reported that low-carbohydrate diet had significantly greater weight loss than calorie-restricted diet over 12 months intervention. Furthermore, Shai[17] compared the effectiveness of weight loss diets among 322 moderately obese subjects and also found that low-carbohydrate diet would be more effective than calorie-restricted(low-fat) diet over 24 months intervention. Therefore, the reported effects of low-carbohydrate diet versus calorie-restricted diet on weight loss have varied.

However, previous studies have examined the effects of low-carbohydrate, energy deficit diet and calorie-restricted, low-fat diet on weight loss, but most of these trials did not compare the weight loss effects of a calorie-restricted diet alone (calorie-restricted, high/moderate-carbohydrate) with a low-carbohydrate diet alone (low-carbohydrate, non-calorie-restriction). In addition, these studies did not provide evidence that a combination of low-carbohydrate and calorie-restricted diet was more effective for weight loss and reducing metabolic risk factors than calorie-restricted diet or low-carbohydrate diet alone. Therefore, the effects of low-carbohydrate and calorie-restricted diet on weight loss and metabolic risk factors in overweight/obese adults need to be tested in future clinical studies.

2. Specific Aims

In this study, we aim to test the effects on weight loss in overweight/ obese adults by a combination of LC diet and CR diet. We propose to conduct a 12-week clinical trial to evaluate the effects of or LC and CR diet on weight loss. We hypothesize that a combination of LC and CR diet would have a greater weight loss and favorable metabolic effects than LC diet and CR diet alone in overweight/obese adults.

The primary specific aims are:

2.1. To test the effect of LC+CR diet over 12 weeks on changes in BMI compared to LC diet and CR diet alone;

2.2. To test the effect of LC diet over 12 weeks on changes in BMI compared to CR diet;

2.3. To test the effect of LC+CR diet, LC diet and CR diet alone over 12 weeks on changes in BMI compared to the NC group.

The secondary specific aim is:

2.4. To compare the effect of LC+CR diet, LC diet and CR diet control on waist circumference, body fat, and metabolic risk factors.

3. Research Design and Methods

3.1. Overall designs

This study is a randomized, multiple-center, parallel group, controlled clinical trial. After screening, all eligible trial participants will be randomly assigned to NC

group, LC diet group, CR diet group and LC+CR diet group (**Figure 1**) for 12 weeks with an allocation ratio of 1:1:1:1. A total of 300 participants will be enrolled. The study will be conducted in the following 13 hospitals in Guangdong Province, China:

- Zhujiang Hospital, Southern Medical University, Guangzhou;
- Huizhou Central People's Hospital, Huizhou;
- Qingyuan People's Hospital, Qingyuan;
- The Third Affiliated Hospital of Guangzhou Medical University, Guangzhou;
- DONGGUAN KANGHUA HOSPITAL, Dongguan;
- Hexian Memorial Affiliated Hospital of Southern Medical University, Guangzhou;
- Guangdong Provincial People's Hospital, Guangzhou.
- Guangdong Second Province Central Hospital, Guangzhou;
- Dongguan People's Hospital, Dongguan;
- Affiliated Hospital of Guangdong Medical University, Zhanjiang;
- The First People's Hospital of Shunde, Shunde;
- The Eighth Affiliated Hospital, Sun Yat-Sen University, Shenzhen;
- Shaoguan First People's Hospital, Shaoguan.
- **Figure 1. The schedule for screening**

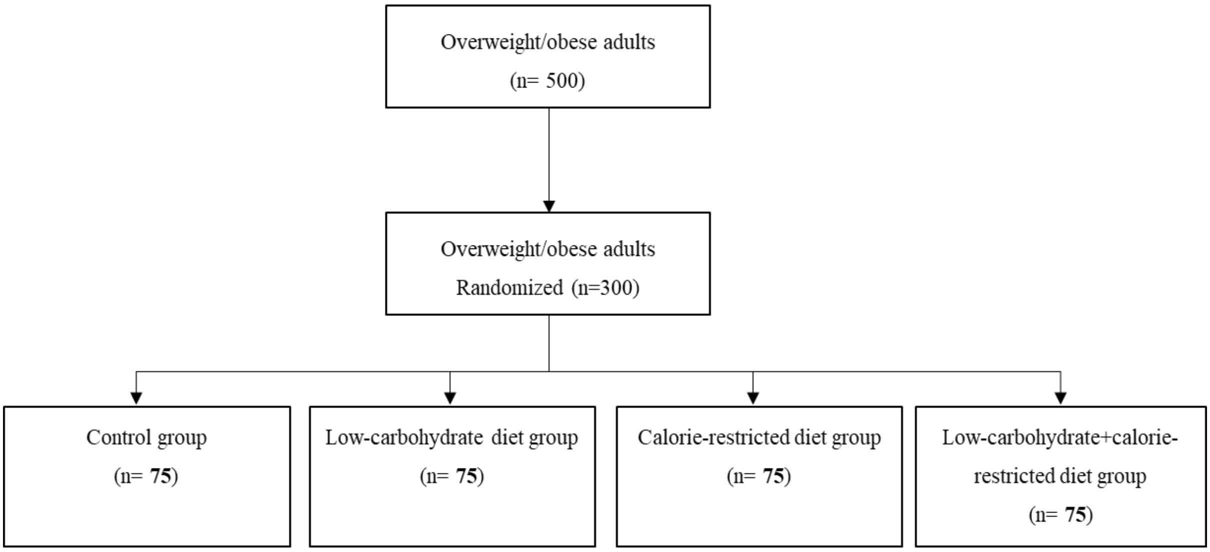

3.2. Statistical power and sample size

The study will enroll 300 participants, who will be randomly allocated to four groups with an equal number of individuals per group:

- NC group
- LC diet group
- CR diet group
- LC+CR diet group

The primary outcome (BMI) will be compared between any two groups at 12 weeks. The sample size calculation was based on the following assumptions:

- Statistical power 85%
- Significance level of 0.05 for the Bonferroni correction of multiple comparisons using a 2-tailed test
- Detectable effect size of BMI = -0.6 kg/m^2[25-28]
- Standard deviation of BMI = -0.6 kg/m^2[25-28]

Thus, 60 subjects in each group are required. Assuming a dropout rate of 20%, we will recruit 300 participants and randomly allocate 75 subjects to each group in the proposed clinical trial.

4. Inclusion and Exclusion Criteria

4.1. Inclusion criteria

- BMI≥24kg/m^2;
- 18 to 65 years old;

4.2. Exclusion criteria:

- Having any medical condition that would affect metabolism (i.e. diabetes, known hyperthyroidism or hypothyroidism);
- Myocardial infarction in the past six months;
- Heart failure (New York Heart Association III or IV);
- Uncontrolled hypertension (i.e. SBP>180 mmHg, and/or DBP >100 mmHg);
- Liver damage: total bilirubin >1.5× ULN(>34.2μmol/L(>2mg/dL) or AST and ALT >3× ULN (150U/L);
- Chronic kidney disease (serum creatinine >=1.5 mg/dL in men and >=1.3 mg/dL in women);
- Subjects suffered from acute or chronic gastrointestinal symptoms or had a history of gall bladder disease or biliary obstructive diseases in the past 30 days;
- Those allergies of proteins such as beans, wheat, milk;
- Taking medication that would affect metabolism or weight loss (i.e. thyroid

medication and glucocorticoids) or anticipating a weight-loss program;

- Participating in weight loss programs currently or in the past 3 months;
- Currently pregnant or planning to be pregnant;
- Unable to participate in the follow-up examination.

5. Recruitment

Participants will be recruited from Guangdong province, China, from April, 2018. A total of 500 overweight/ obese adults aged 18-65 years old (BMI≥24kg/m^2) from previous community screening programs will be invited to the near sub-center. Once a patient is confirmed to be eligible, the clinician will review the study procedures and requirements with the patient and discuss his or her commitment to the study and acceptance of randomization. Written informed consent will be obtained from each participant, and a baseline evaluation will be performed at the next visit, which is immediately scheduled by the clinician assistant.

6. Randomization and Masking

6.1. Randomization

Randomization will be conducted by Department of Bio-Statistics, Southern Medical University, China. Patients will be randomly assigned to NC group, LC diet group, CR diet group or LC+CR diet group using a block design. The randomization scheme is generated and concealed until an eligible participant is ready to be randomized. Prior to randomization, the study coordinator should confirm that all screening procedures have been completed, the participant meets all eligibility criteria, and all required baseline data have been collected. Any individual lacking required documents and data will not be randomized.

6.2. Masking

This is an open-label trial.

7. Intervention

This trial aims to test the effects of different diets on weight loss and metabolic risk factors. The duration of the intervention is 12 weeks.

7.1. Intervention programs

- **NC group**

Participants in the NC group were designed to follow a non–restricted-calorie

diet with 55-65% of calories from carbohydrate, 30% from fat and 20% from protein[19, 31–32]. Participants will not be food choice restricted but they should avoid overeating. No specific exercise program will be recommended.

- **LC group**

Participants assigned to the LC diet group were instructed to follow a non–restricted-calorie diet with less than 26% of calories from carbohydrates, 50% from fat and more than 24% from proteinc[15, 29-30]. Participants will receive free standard LC replacement, a nutritional bar (Nanda Fit Nutrition and Health Consulting Co., Ltd, Guangzhou, China) both at lunch and dinner (370kcal totally, specific formula shown in **Table 1**). Participants will not be food choice restricted but they should avoid overeating. Participants will be instructed to drink 2L water/day and to minimize fruits, bread, added sugars, refined white flour products, and sources of trans fats. No specific exercise program will be recommended.

- **CR group**

Participants in the CR diet group were restricted daily calorie to 1,200-1,500kcal with 55-65% of calories from carbohydrate, 30% from fat and 20% from protein[29-30,32]. Participants will not be food choice restricted. No specific exercise program will be recommended.

- **LC+CR group**

Participants assigned to LC+CR diet group were instructed to follow a daily calorie intake of 1,200-1,500kcal with less than 26% of calories from carbohydrate, 50% from fat and more than 24% from protein[29-31]. Participants will receive free standard LC replacement, a nutritional bar (Nanda Fit Nutrition and Health Consulting Co., Ltd, Guangzhou, China) at lunch and dinner (370kcal totally, specific formula shown in **Table 1**). Participants will not be food choice restricted and will be instructed to drink 2L water/day and to minimize fruits, bread, added sugars, refined white flour products, and sources of trans fats. No specific exercise program will be recommended.

Table 1. The Components of the Nutrition Food Bar

Content	Per 100 grams	NRV%
Energy	1723KJ	20
Protein	32.2g	54
Fat	15.7g	26

Trans Fatty Acids	0g	/
Carbohydrate	29.2	10
Meal Fiber	12.7g	51
Sodium	65mg	3

Master formula: whey protein, soy protein isolate, chia seed, oligosaccharide, Collagen, konjac extract, γ-aminobutyric acid.
NRV, nutrient reference values

- **Health education**

All the participants will meet in weekly counseling sessions by dietitians to provide dietitian plans and give advice for 12 weeks. Guidance on counting macronutrients, reading nutrition labels and detailed dietary recipes will be given to four groups. All study participants were instructed to record macronutrients, nutrition labels and detailed dietary recipes all they consume whole day for 3 days per week, including 2 working days and 1 weekend day. Nutrient intake will be calculated based on the nutrient content listed in the Chinese Food Composition Table[33]. Furthermore, trial participants will be required to wear a Mi Band 2 Smart Bracelet (Xiaomi, Beijing, China) during the whole experimental process to record their regular physical activity. They were advised to do regular exercise 3 times a week with durations for 30 minutes. During each follow-up visit, participants will be asked about adverse reactions such as hunger, fatigue, palpitation, anorexia, constipation, alopecia. Research assistants will meet participants weekly to assess their compliance and provide advice on adherence to the diet program.

7.2. Intervention monitoring and quality control

All the food consumption and exercise will be supervised by research assistants. The types of foods, amount of food and cooking methods, will be recorded by participants themselves. Nutrient intake will be estimated every 4 weeks, at baseline and 4-, 8- and 12-week. In order to reduce impacts caused by exercise, participants will be instructed not to change their daily physical activity and record their regular physical activity by wearing a Mi Band 2 Smart Bracelet (Xiaomi, Beijing, China) and record macronutrients, nutrition labels and detailed dietary recipes. Research assistants will call participants twice weekly to assess their compliance and provide advice on adherence to the dietary program. Adherence to the dietary program was

defined according to the number of days that a participant met the requirements of the assigned diet.

8. Study Outcomes

8.1. Primary outcome:

Changes in BMI from baseline at 12 weeks after intervention.

8.2. Secondary outcomes:

- Changes in body weight, waist circumference, WHR and body fat
- Changes in lipid and glucose
- Changes in insulin resistance
- Changes in urinary albumin and liver enzyme

9. Participant Termination and Retention

9.1. 9.1 Termination criteria

- Adverse events or other unexpected reasons (e.g., hypoglycaemia, constipation, gastrointestinal discomfort, etc.)
- Inability to complete required dietary program
- Unwillingness to be followed-up

9.2. Study participants retention

The study participant retention is critically important and every effort will be made to increase participants' adherence to their intervention and follow-up visit schedule. At weekly staff meetings, recruitment and retention will be discussed. Visits will be scheduled at the convenience of the patients. Personalized birthday, holiday, and anniversary cards will be sent to the participants. Small gifts will be used to improve participants' connection to the study and research staff.

10. Data collection

10.1. Measurements

1). Medical history: personal information (age, gender, marital status, occupation, education, household income, etc.), medical history including medications, and lifestyle risk factors (cigarette smoking, alcohol drinking, physical activity, and dietary habits).

2). Anthropometric measurements: height, weight, waist circumference, hip

circumference.

3). Biochemical measurements: fasting plasma glucose, serum triglycerides, total cholesterol, LDL-C and HDL-C, liver enzymes, and uric acid.

4). Insulin sensitivity: HOMA-IR.

5). Body composition will determine by IOI353 Body Composition Analyser (Jawon Medical, Gyeongsansi, South Korea).

10.2. Methods

A. Questionnaire

Demographic information: A self-report demographic questionnaire will be used to collect participant characteristics at baseline, including age, race/ethnicity, socioeconomic status (employment, education, and income), internet usage, and health status.

Medical history: Medical history including medication used will be self-reported by participants at the screening visit and subsequent follow-up visits. The medical recordings will be reviewed at times to ensure all the data are complete.

Diet: Three 24-hour dietary recalls will be conducted at baseline and during follow-up visits. Nutrient intake will be calculated using the China Food Composition table.

Physical activity: Physical activity will be recorded for 3 continuous days by Mi Band 2 Smart Bracelet (Xiaomi, Beijing, China).

B. Clinical examination

B1. Weight, waist circumference, and blood pressure

Height will be measured to the nearest 0.5 cm at baseline and follow-up visits using a wall-mounted stadiometer. Body weight will be measured to the nearest 0.1 kg in duplicate at each visit using a digital scale with participants dressed in light indoor clothes and without shoes. A gulick tape measure will be used to obtain duplicate measurements of waist and hip girths to the nearest 0.1 cm. Blood pressure will be measured in triplicate at each visit using the Omron HEM-907-XL automated sphygmomanometer. Participants will avoid exercise, smoking, coffee or tea for 30 minutes. Participants will be seated quietly for 5 minutes before measurement.

B2. Body composition

The bioelectrical impedance method will be used to measure body fat content using an IOI353 Body Composition Analyzer(Jawon Medical, Gyeongsansi, South

Korea) to evaluate the body fat, body muscle.

C. Biochemical tests

Overnight blood samples and spot urine will be collected to measure metabolic risk actors (plasma glucose, insulin, triglycerides, total cholesterol, LDL- and HDL-cholesterol, liver enzymes, and uric acid) using standard methods at the clincial laboratory of each subcenter.

11. Baseline and Follow-up Visits

11.1. Screening visit (V0)

A. Questionnaire

Demographic information: age, gender, education.

Medical history: personal history of acute or chronic viral hepatitis, drug-induced liver diseases, autoimmune hepatitis, Cerebral infarction, cancer, myocardial infarction, hyperthyroidism, hypothyroidism, and diabetes, or having a medical condition or taking medication that would limit metabolism or weight loss.

Medications: lipid-lowering, anti-hypertension, and liver-protection medication (specific drugs dosage and termination time).

Lifestyle risk factors: alcohol drinking and cigarette smoking.

B. Physical examination

Height, weight, waist circumference, hip circumference, and blood pressure;

C. Laboratory examination

Regular blood and urine tests

Liver enzymes

Serum triglycerides, total, LDL-C and HDL-C, and plasma glucose, insulin, uric acid

D. Other examinations

Electrocardiograph (ECG)

Body composition analyzation for body fat

11.2. Randomization visit (V1)

A. Questionnaires

Assessment of three 24-hour dietary recalls

Assessment of baseline physical activity (for 3 continuous days)

B. Measurements of the primary and secondary outcomes

anthropometric examination.

Measurement of body fat distribution by Body composition analyzer(Jawon Medical, Gyeongsansi, South Korea)

C. Collection of blood

D. Randomization:

Participants will be allocated into the NC group, LC diet group, CR diet group and LC+CR diet group using a computer program.

11.3. Week 0 visit (V2)

A. Exercise and dietary records will be reviewed.

B. Dietary evaluation and adjustment:

Participants will be asked to be as specific as possible about the type and amount(g/ml) of food and beverage consumed (e.g. skimmed or non-skimmed milk, 200ml), including type of cooking method used (e.g. roasted, boiled, etc.) by a 24-hour dietary recalls (3 days including two working days and one weekend day). Dietitians estimate and record the total intakes of calories(kcal), protein (%), fat (%), carbohydrates (%) according to the China Food Composition table.

C. Height, body weight, waist circumference, hip circumference, will be recorded at the end of every month.

11.4. Week 4 visit (V3)

A. Questionnaires

Assessment of three 24-hour dietary recalls

Assessment of baseline physical activity (for 3 continuous days)

B. Physical examination: height, weight, waist circumference, hip circumference, body fat

C. Laboratory examination:

Routine tests for blood, serum lipids, liver enzymes, uric acid, fasting plasma glucose, serum insulin

D. Evaluation and adjustment of dietary.

11.5. Week 8 visit (V4)

A. Questionnaires

Assessment of three 24-hour dietary recalls

Assessment of baseline physical activity (for 3 continuous days)

B. Physical examination: height, weight, waist circumference, hip circumference, body fat

C. Laboratory examination:

Routine tests for blood, serum lipids, liver enzymes, uric acid, fasting plasma glucose, serum insulin

D. Evaluation and adjustment of dietary.

11.6. Week 12 visit (V5)

A. Questionnaires

Assessment of three 24-hour dietary recalls

Assessment of baseline physical activity (for 3 continuous days)

B. Physical examination: height, weight, waist circumference, hip circumference, body fat

C. Laboratory examination:

Routine tests for blood, serum lipids, liver enzymes, uric acid, fasting plasma glucose, serum insulin

D. Evaluation and adjustment of dietary.

11.7. Monitoring and quality control

A. Dietitians and nurses will receive training on data collection or intervention procedures.

B. Exercise and dietary will be supervised by study staff.

C. Compliance to exercise will be recorded by a Mi Band 2 Smart Bracelet (Xiaomi, Beijing, China) and accessed by study staff weekly.

D. Plasma glucose, lipid, and liver enzymes will be determined at the each subcenter with stringent quality control.

E. All study outcomes will be measured by trained staff using a stringent quality control process. Participants will be informed not to eat, smoke, drink, or do any strenuous exercise at least 10 hours before the examination at each visit.

12. Safety and Adverse Events

12.1. Safety measures and laboratory examinations

All participants will be evaluated for their pulse rate and blood pressure before intervention and will be evaluated for any physical discomfort, including eart palpitations, chest pain, constipation, gastrointestinal discomfort and hypoglycaemia

during intervention. The occurrence, severity, and duration of physical discomfort will be recorded.

12.2. Definition of adverse events

All medical adverse events will be recorded and the causal relationship to the exercise intervention will be further evaluated. The relevant adverse events are defined as heart palpitations, chest pain, constipation, gastrointestinal discomfort and hypoglycaemia during intervention.

12.3. Recording of adverse events

Any medical adverse events, including physical discomfort or abnormal laboratory examinations, will be recorded. Additionally, the nature, severity, and causal-effect relationship will be evaluated by physicians and recorded in a timely manner on case report forms.

12.4. Definition of a causal-effect relationship

With regard to the causal-effect relationship, medical adverse events are divided into five categories: definite, probable, possible, unlikely, and unrelated. Only "definite” and “probable” are counted as adverse events.

12.5. Assessment of severity

The severity of adverse events is categorized at three levels as follows:

Mild: usually temporary and not affecting daily activities.

Moderate: causing discomfort and affecting daily activities, though tolerable and not requiring participants to take any medication.

Severe: disrupting daily activities and intolerable, requiring participants to take medication immediately.

Treatment Physicians will observe and record the progress of adverse events and keep track of those participants who have dropped out until the adverse events have completely subsided. Additionally, physicians will evaluate those adverse events for a causal-effect relationship.

12.6. Severe adverse events

Definition:

According to the definition by the Food and Drug Administration, USA, severe adverse events are defined as any of the following:

Life-threatening experience

Death:

Inpatient hospitalization or prolongation of existing hospitalization

A persistent or significant disability/incapacity

A congenital anomaly/birth defect

Reporting of severe adverse events

If severe adverse events occur in participants, physicians should immediately provide the appropriate care to ensure their safety. Additionally, physicians will report the adverse events and treatment to the Principal Investigator and the Committee within 24 hours and complete the report form.

12.7. Safety methods

When an adverse reaction is discovered, the researcher can take necessary treatment measures, such as medication or surgical intervention, according to the situation, and decide whether to suspend the trial. In the event of a serious adverse event, the unit undertaking the trial and research must take necessary measures to protect the safety of the subjects.

13. Drop-outs

13.1. Dropout determination

Participants who withdraw from the trial for any reason will be considered a drop-out.

13.2. Common reasons for dropping out

- Poor compliance with the protocol
- Adverse events
- Lack of efficacy
- Withdraw and quit
- Others

13.3. Dropout management

Physicians should complete the case report form and record the reason for dropping out. Physicians should also assess the causal-effect relationship of the intervention with adverse events and collect detailed data related to the last exercise session. The data from subjects who have dropped out will be stored and used for intention-to-treat analysis

14. Ethical requirements

The study followed the ethical guidelines of the Helsinki Declaration and obtained the approval of the Ethics Committee of Zhujiang Hospital of Southern Medical University (ratification No.:2018-NFMDXK-001), the host research unit, and complete registration at the China Clinical trial Registration Center (registration number: ChiCTR1800015156).

15. Data management

15.1. Case report form

All case report forms for each participant should be filled out by study staff in a timely manner. The case report form should be double-checked for potential errors or missing data prior to patients leaving the clinic. All data, including screening assessments, questionnaires, physical examinations, and laboratory examinations, will be filed in the participant's chart. Original documents, participants' charts, and CRF forms will be stored in the study office

15.2. Data entry

All data will be double-entered by researcher staff. Two sets of databases will be generated and tested for consistency using the SAS program. Whenever inconsistencies are found, the data will be corrected by re-examination of the original case report forms or laboratory reports.

15.3. Data reports

Several standardized reports will be generated as follows: 1) participant recruitment and follow-up; 2) demographics; 3) data quality and monitoring; 4) adverse events. These reports will be used for study management. These reports will be blinded to research personnel who collect study outcomes.

16. Data Analysis Plan

Data will be analyzed according to participants' randomization assignments, regardless of their subsequent status (intention-to-treat).

16.1. Study outcomes

The primary outcome is change of body mass index (BMI). The secondary outcomes included changes in body weight, body fat, waist circumference and metabolic risk factors.

16.2. Analysis methods

Data will be analyzed according to participants' randomization assignment (intent-to treat). A mixed-effects model will be used to assess the effects of diet programs on the change in BMI and an autoregressive correlation matrix will be used to correct within-participant correlation for repeated measurements. In this model, variables will be assumed to be random effects, and intervention group, time, and their 2-factor interactions, age, sex will be assumed to be estimable fixed effects. PROC MIXED of SAS statistical software, version 9.4 (SAS Institute Inc), will be used to obtain point estimates and SEs of the treatment effects and to test for differences between treatments. Multiple imputations for missing data in the multivariable analyses will be conducted using the Markov chain Monte Carlo method. P-value <0.05 will be considered as statistically significance. Group differences in the study outcomes will be evaluated using the general linear model for continuous variables and the chi-squared test for categorical variables. Data will be presented as least-squares means with 95% confidence intervals (CIs).

17. Training for Study Staff

All study staff (physicians and nurses) will be trained and certified before the study is initiated. The training program will include instruction on questionnaire administration and anthropometrics (height, weight, waist circumference, and hip girth) and blood pressure measurement. A standard questionnaire will be used, which includes personal information (name, sex, age, place of birth, marital status, occupation, education, household income, etc.), medical history, medication use, family history, lifestyle factors (smoking and drinking), physical activity, dietary habits, and menstrual and reproductive history. Anthropometric measurements and blood pressure will be obtained using a standard protocol. The data, including that for dietary record, duration, heart rate, and anthropometric measurements, will be collected and recorded in CRFs by research assistants every week. Body fat mass will be quantified using a body composition analyzer (Jawon Medical, Gyeongsansi, South Korea). All technologists are experienced and blind to randomization of the participants.
