# Supplementary material for: The effect of dietary carbohydrate and calorie restriction on weight and metabolic health in overweight/obese individuals: a multi-center randomized controlled trial
Source: BMC Med. 2023 May 24;21:192. doi: 10.1186/s12916-023-02869-9 (PMC10210464; doi:10.1186/s12916-023-02869-9)
Supplement: Supplementary file 2 — Additional file 2: Fig. S1. Adherence to the prescribed diets over 12 weeks. Fig. S2. Metabolites changes of LC diet and CR diet at baseline and 12 weeks. Table S1. Food profile of diet interventions. Table S2. Baseline characteristics of study participants included in completer analysis. Table S3. Daily physical activityat baseline and during follow-up. Table S4. Energy and nutrition intake during follow-up of completer participants. Table S5. Adherence during the 12-week intervention. Table S6. Effects of dietary intake on weight loss and body fat after -week intervention of completer participants. Table S7. Effects of dietary intake on cardiovascular risk factors during 12-week intervention of completer participants. Table S8. Tests of between subject-effect. [file 12916_2023_2869_MOESM2_ESM.pdf]

Supplementary Appendix

Supplemental Tables and Figures Pages **page**

Fig. S1. Adherence to the prescribed diets over 12 weeks 1
Fig. S2. Metabolites changes of LC diet and CR diet at baseline and 12 weeks 2
Table S1. Food profile of diet interventions 3
Table S2. Baseline characteristics of study participants included in completer analysis 4
Table S3. Daily physical activity (steps/day) at baseline and during follow-up 6
Table S4. Energy and nutrition intake during follow-up of completer participants 7
Table S5. Adherence during the 12-week intervention 9
Table S6. Effects of dietary intake on weight loss and body fat after 12-week intervention of completer participants 10
Table S7. Effects of dietary intake on cardiovascular risk factors during 12-week intervention of completer participants 11
Table S8. Tests of between subject-effect 13

Fig. S1

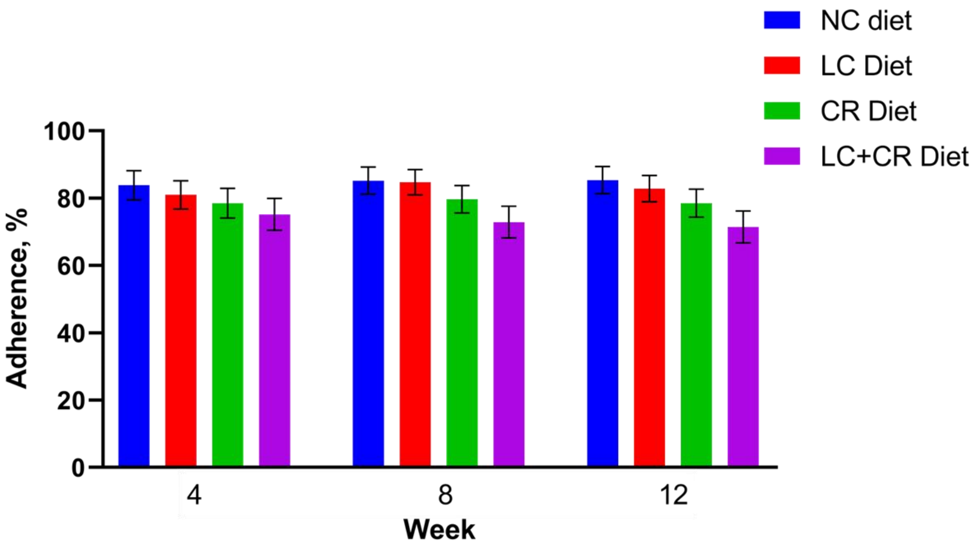

No. of Participants			
NC Diet	65	63	62
LC Diet	71	67	67
CR Diet	67	64	63
LC+CR Diet	69	69	69

Adherence to the prescribed diets over 12 weeks. Data presented are observed data for the full analysis set from the in-trial period. Error bars represent standard error for the mean. Participant numbers shown denote those contributing to the mean.

Fig. S2

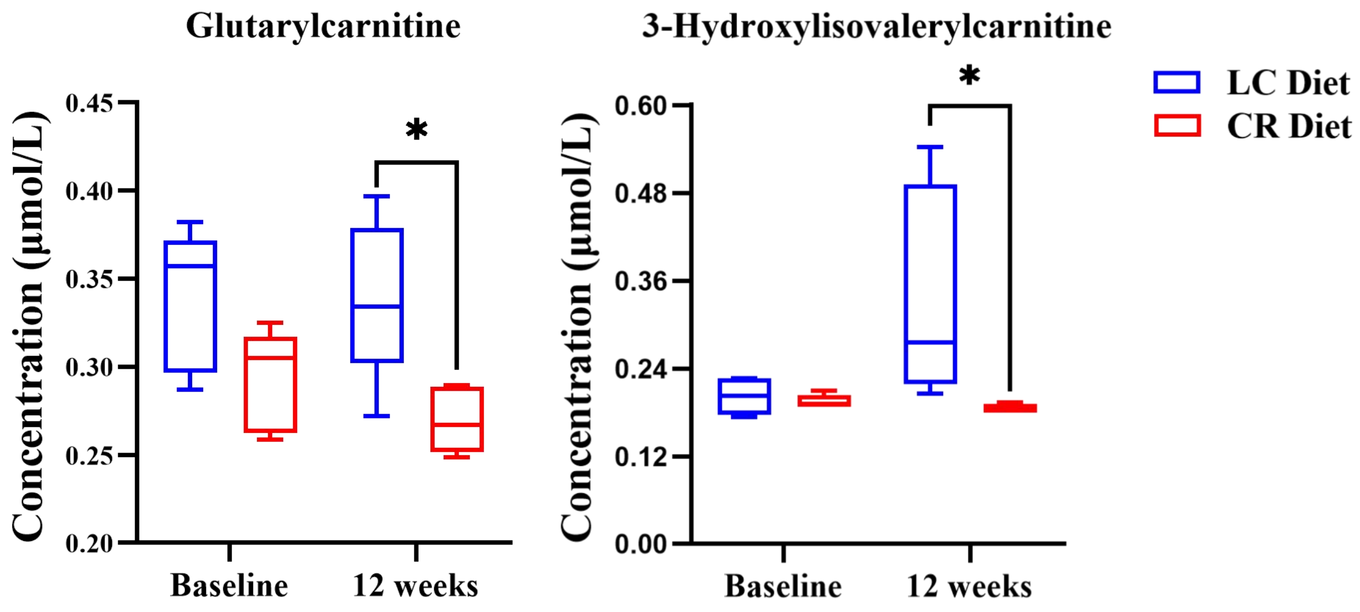

Metabolites changes of LC diet and CR diet at baseline and 12 weeks. *denotes the significant differences of metabolites between LC diet and CR diet.

Table S1. Food profile of diet interventions

NC diet, 1700-1800kcal	LC diet, 1700-1800kcal	CR diet, 1200-1300kcal	LC+CR diet，1200-1300kcal
• Staple 800kcal<br>(e.g., rice 700g or noodles 250g or pancakes 310g or potatoes/corn 920g)	• 2 nutritional bar* 370kcal	• Staple 520kcal<br>(e.g. rice 450g or noodles 162.5g or pancakes 201.5g or potatoes/corn 598g);	• 2 nutritional bar* 370kcal
• Vegetables 300~500g;<br>• Fruits 90kcal<br>(e.g., 1 apple/mango)	• Low starch vegetables 400~500g;<br>• Low GI# fruits 180kcal<br>(e.g., 2 apples/orange/peach or 20 grapes）	• Vegetables 300~500g;<br>• Fruits 90kcal<br>(e.g., 1 apple/ 1 mango）	• Low starch vegetables 400~500g;<br>• Low GI# fruits 90kcal<br>(e.g., 1apples/orange/peach or 10 grapes）
• Meat 350kcal<br>(e.g., lean pork/red meat 250g or fish/shrimp 350g);<br>• 1 egg；<br>• 200ml milk or 400ml soy milk	• Meat 430kcal<br>(e.g., pork/red meat 300g or fish/ shrimp 430g);<br>• 2 eggs；<br>• 250ml milk or 500ml soy milk	• Meat 215kcal<br>(e.g., lean pork/red meat 150g or fish/ shrimp 215g);<br>• 1 egg；<br>• 200ml milk or 400ml soy milk;	• Meat 300kcal<br>(e.g., lean pork/red meat 200g or fish/ shrimp 300g）；<br>• 1 egg；<br>• 200ml milk or 400ml soy milk
• Nut 55kcal<br>(e.g., walnuts / apricot kernel 10g)	• Nut 110kcal<br>(e.g., walnuts / apricot kernel 20g)	• Nut 55kcal<br>(e.g., walnuts / apricot kernel 10g)	• Nut 55kcal<br>(e.g., walnuts / apricot kernel 10g)
• Cooking oil 180kcal<br>(e.g., peanut oil / canola oil / soybean oil 20g)	• Cooking oil 270kcal<br>(e.g., peanut oil / canola oil / soybean oil 30g)	• Cooking oil 135kcal<br>(e.g., peanut oil / canola oil / soybean oil 15g)	• Cooking oil 180kcal<br>(e.g., peanut oil / canola oil / soybean oil 20g)

* 45g each nutritional bar providing energy of 185 kcal, including32.2g protein, 15.7g fat and 29.2g carbohydrate. Main components: whey protein, soy protein isolate, chia seed, oligosaccharide, collagen, konjac extract, γ-aminobutyric acid.

#GI, glycemic index

Table S2. Baseline characteristics of study participants included in completer analysis

Characteristic	NC diet (n =62)	LC Diet (n = 67)	CR Diet (n = 63)	LC+CR Diet (n = 69)
Female, No. (%)	40(64.5)	50(74.6)	45(71.4)	50(72.5)
Age, y	35.2 (8.5)	34.6 (7.9)	33.4 (6.1)	33.4 (6.8)
High school education, No. (%)	11(17.7)	22(34.3)	16(25.4)	12(17.4)
Current cigarette smoking, No. (%)	10(16.1)	12(17.9)	15(23.8)	11(16.0)
Current alcohol drinking, No. (%)	20(32.3)	12(17.9)	29(46.0)	11(16.0)
Total energy intake, kcal/d	1806.7 (186.9)	1923.3 (336.8)	1825.0 (250.0)	1921.7 (433.1)
Carbohydrate intake, %	50.0 (6.7)	48.7 (5.3)	50.3 (3.8)	54.2 (7.6)
Protein intake, %	21.0 (4.6)	22.0 (3.0)	19.0 (3.4)	18.2 (4.6)
Fat intake, %	29.0 (3.2)	29.3 (6.7)	30.8 (2.9)	27.7 (3.7)
Weight, kg	78.7 (12.7)	78.7 (13.0)	78.7 (12.1)	83.3 (19.3)
BMI, kg/m^2	29.6 (3.5)	30.0 (3.6)	30.0 (3.3)	31.0 (4.8)
Waist circumference, cm	93.7 (9.4)	93.3 (9.6)	94.3 (9.0)	95.2 (13.7)
WHR	0.89 (0.6)	0.89 (0.6)	0.89 (0.6)	0.89 (0.7)
Heart rate, beats /min	76.7 (8.2)	78.2 (8.6)	77.9 (11.7)	77.0 (8.8)
Blood pressure, mm Hg				
Systolic	119.0 (12.8)	114.8 (10.1)	114.4 (10.7)	115.0 (12.1)
Diastolic	75.2 (17.2)	76.6 (9.5)	78.7 (7.4)	78.1 (7.6)
Plasma glucose, mg/dL	94.1 (9.3)	91.1 (9.7)	94.2 (9.0)	93.9 (8.3)
HOMA-IR	3.4 (2.5)	3.2 (2.3)	3.3 (2.9)	3.8 (2.5)
Serum triglycerides, mg/dL	195.5 (36.4)	189.0 (39.4)	197.8 (39.0)	192.8 (37.0)
Serum total cholesterol, mg/dL	140.2 (75.5)	133.1 (85.0)	134.9 (100.3)	155.0 (122.3)
HDL, mg/dL	46.6 (12.0)	48.9 (13.6)	47.6 (9.4)	45.3 (10.4)
LDL, mg/dL	119.9 (33.1)	113.4 (34.6)	123.0 (30.8)	116.7 (26.6)

Characteristic	NC diet (n =62)	LC Diet (n = 67)	CR Diet (n = 63)	LC+CR Diet (n = 69)
ALT, IU/L	28.4 (22.7)	24.0 (14.8)	27.0 (19.6)	28.0 (20.3)
AST, IU/L	22.2 (10.0)	19.9 (7.9)	20.4 (9.1)	20.4 (8.4)
Uric acid, mg/dl	6.7 (1.4)	6.4 (1.6)	6.8 (1.7)	6.7 (2.0)
Body fat, %	34.2 (4.5)	34.8 (4.9)	35.1 (4.3)	35.2 (4.0)
Visceral Fat Area, cm^2	108.0 (36.8)	95.7(33.2)	103.8(29.1)	92.5(42.3)
Body muscle rate, %	60.6 (4.3)	59.6 (4.5)	59.4 (4.1)	59.4 (3.8)

Table S3. Daily physical activity (steps/day) at baseline and during follow-up

Time	NC diet (n = 74)	LC Diet (n = 76)	CR Diet (n = 72)	LC+CR Diet (n = 76)	*P* Values			
					LC diet vs NC diet	LC Diet vs CR Diet	LC+CR Diet vs LC Diet	LC+CR Diet vs CR Diet
Baseline	7614(2100)	7392(2406)	7107(2488)	7248(2702)	0.584	0.483	0.718	0.728
4 weeks	8010(2672)	7356(2310)	6875(2436)	7170(2139)	0.106	0.237	0.64	0.468
8 weeks	7205(2534)	7412(2531)	7285(2832)	7759(2499)	0.609	0.756	0.383	0.244
12 weeks	6745(2241)	7443(2800)	7036(2464)	7612(2330)	0.084	0.318	0.671	0.157

Data are means (SD).

Table S4. Energy and nutrition intake during follow-up of completer participants

	Changes (95% CIs)				*P* Values				
**Characteristic**	**NC diet (n = 62)**	**LC Diet (n = 67**	**CR Diet (n = 63)**	**LC+CR Diet (n = 69)**	**LC diet vs NC diet**	**LC+CR Diet vs CR Diet**	**CR diet vs NC diet**	**LC+CR Diet vs LC Diet**	**LC Diet vs CR Diet**
Energy intake, kcal									
Week 4	1660.6(1588.6 to 1732.7)	1517.8(1453.2 to 1582.3)	1218.4(1147.9 to 1288.8)	1271.1(1205.4 to 1336.9)	0.004	0.282	<0.001	<0.001	<0.001
Week 8	1655.0(1582.6 to 1727.3)	1507.8(1442.9 to 1572.7)	1221.8(1151 to 1292.6)	1271.8(1205.7 to 1337.9)	0.003	0.310	<0.001	<0.001	<0.001
Week 12	1639.3(1567 to 1711.6)	1500.4(1435.6 to 1565.1)	1220.1(1149.4 to 1290.8)	1266.5(1200.5 to 1332.5)	0.005	0.345	<0.001	<0.001	<0.001
Carbohydrate intake, g									
Week 4	206.4(196.4 to 216.4)	62.5(53.5 to 71.5)	145.5(135.7 to 155.2)	56.0(46.8 to 65.1)	<0.001	<0.001	<0.001	0.315	<0.001
Week 8	205.0(194.5 to 215.5)	62.4(53 to 71.8)	146.5(136.3 to 156.8)	56.3(46.7 to 65.9)	<0.001	<0.001	<0.001	0.374	<0.001
Week 12	201.8(191.3 to 212.3)	63.4(54 to 72.8)	145.3(135.0 to 155.6)	56.9(47.4 to 66.5)	<0.001	<0.001	<0.001	0.343	<0.001
Carbohydrate intake, %									
Week 4	49.4(48.0 to 50.8)	16.6(15.3 to 17.8)	47.5(46.2 to 48.9)	17.6(16.4 to 18.9)	<0.001	<0.001	0.058	0.233	<0.001
Week 8	49.2(47.7 to 50.6)	16.6(15.3 to 17.9)	47.8(46.4 to 49.2)	17.7(16.4 to 19.1)	<0.001	<0.001	0.172	0.223	<0.001
Week 12	48.8(47.3 to 50.3)	17.1(15.7 to 18.4)	47.4(46 to 48.9)	18.0(16.7 to 19.4)	<0.001	<0.001	0.185	0.328	<0.001
Protein intake, g									
Week 4	37.4(34.6 to 40.2)	55.2(52.6 to 57.7)	27.5(24.7 to 30.2)	47.1(44.5 to 49.6)	<0.001	<0.001	<0.001	<0.001	<0.001
Week 8	37.0(34.2 to 39.7)	55.3(52.8 to 57.8)	28.1(25.4 to 30.8)	46.9(44.4 to 49.5)	<0.001	<0.001	<0.001	<0.001	<0.001
Week 12	36.4(33.6 to 39.1)	55.5(53.0 to 57.9)	28.1(25.4 to 30.8)	46.6(44.1 to 49.1)	<0.001	<0.001	<0.001	<0.001	<0.001
Protein intake, %									
Week 4	20.2(19.0 to 21.5)	32.8(31.6 to 33.9)	20.4(19.1 to 21.6)	33.2(32.0 to 34.3)	<0.001	<0.001	0.883	0.644	<0.001
Week 8	20.1(18.8 to 21.5)	33.0(31.8 to 34.2)	20.8(19.5 to 22.2)	33.1(31.8 to 34.3)	<0.001	<0.001	0.449	0.918	<0.001
Week 12	19.9(18.5 to 21.4)	33.4(32.1 to 34.7)	20.9(19.5 to 22.3)	33.0(31.7 to 34.3)	<0.001	<0.001	0.351	0.651	<0.001
Fat intake, g									

Characteristic	Changes (95% CIs)				P Values				
	NC diet (n = 62)	LC Diet (n = 67	CR Diet (n = 63)	LC+CR Diet (n = 69)	LC diet vs NC diet	LC+CR Diet vs CR Diet	CR diet vs NC diet	LC+CR Diet vs LC Diet	LC Diet vs CR Diet
Week 4	59.2(55.6 to 62.7)	84.3(81.1 to 87.5)	48.5(45.0 to 51.9)	67.4(64.2 to 70.7)	<0.001	<0.001	<0.001	<0.001	<0.001
Week 8	59.2(56.1 to 62.3)	83.0(80.1 to 85.8)	48.3(45.2 to 51.3)	67.5(64.6 to 70.4)	<0.001	<0.001	<0.001	<0.001	<0.001
Week 12	58.9(56.0 to 61.8)	82.9(80.3 to 85.5)	48.5(45.6 to 51.3)	67.0(64.4 to 69.7)	<0.001	<0.001	<0.001	<0.001	<0.001
Fat intake, %									
Week 4	32.3(31.0 to 33.6)	49.8(48.7 to 51.0)	36.3(35.0 to 37.5)	47.9(46.7 to 49.0)	<0.001	<0.001	<0.001	0.019	<0.001
Week 8	32.5(31.3 to 33.7)	49.5(48.4 to 50.5)	36.0.(34.8 to 37.2)	47.8(46.8 to 48.9)	<0.001	<0.001	<0.001	0.037	<0.001
Week 12	32.8(31.5 to 34.0)	49.9(48.8 to 51.0)	36.2(35.0 to 37.4)	47.7(46.6 to 48.8)	<0.001	<0.001	<0.001	0.007	<0.001

Table S5. Adherence during the 12-week intervention

Characteristic	NC diet	LC Diet	CR Diet	LC+CR Diet	P value					
					LC diet vs NC Diet	CR Diet vs NC Diet	LC Diet vs CR Diet	LC+CR Diet vs LC Diet	LC+CR Diet vs CR Diet	4 groups
Week 4	83.8±34.9	81±35.4	78.5±35.9	75.2±39.5	0.648	0.399	0.688	0.348	0.598	0.562
Week 8	85.2±32.1	84.7±30.7	79.6±32.6	72.9±39	0.935	0.355	0.391	0.042	0.250	0.124
Week 12	85.3±31.7	82.8±31.8	78.5±33	71.5±39.4	0.677	0.263	0.469	0.054	0.240	0.100

Data are presented as the mean ± SD unless otherwise indicated.

Table S6. Effects of dietary intake on weight loss and body fat after 12-week intervention of completer participants

Outcomes	Changes (95% CIs)				*P* Values				
	NC diet (n =62)	LC Diet (n = 67)	CR Diet (n =63)	LC+CR Diet (n = 69)	LC diet vs NC diet	LC Diet vs CR Diet	LC+CR Diet vs LC Diet	LC+CR Diet vs CR Diet	Group differences
BMI, kg/m^2	-0.6(-1.9 to 0.7)	-2.4(-3.6 to -1.1)	-1.2(-2.5 to 0.1)	-3.0(-4.2 to -1.7)	<0.001	<0.001	0.001	<0.001	<0.001
Weight, kg	-1.5(-6.5 to 3.4)	-6.1(-10.8 to -1.3)	-3.1(-8.0 to 1.8)	-8.1(-12.8 to -3.4)	<0.001	<0.001	<0.001	<0.001	<0.001
Waist circumference, cm	-1.7(-5.2 to 1.9)	-5.7(-9.2 to -2.3)	-3.2(-6.8 to 0.3)	-7.1(-10.5 to -3.7)	<0.001	<0.001	0.012	<0.001	<0.001
WHR ratio	-0.01(-0.03 to 0.01)	-0.03(-0.05 to 0.00)	-0.02(-0.04 to 0.00)	-0.03(-0.05 to -0.01)	<0.001	0.192	0.237	0.014	<0.001
Body fat, %	-0.7(-2.3 to 0.9)	-2.5(-4.0 to -0.9)	-1.5(-3.1 to -0.1)	-3.1(-4.7 to -1.6)	<0.001	0.007	0.064	<0.001	<0.001

Table S7. Effects of dietary intake on cardiovascular risk factors during 12-week intervention of completer participants

Outcome	Between-group differences from baseline to week 12						*P* Value of Group differences
	LC diet vs NC diet		CR diet vs NC diet		LC+CR diet vs NC diet		
	Changes (95% CIs)	P Value	Changes (95% CIs)	P Value	Changes (95% CIs)	P Value	
Plasma glucose, mg/dL							
Week 4	-3.1(-6.5 to 0.3)	0.937	-2.4(-5.8 to 1.0)	0.092	-4.0 (-7.4 to -0.6)	0.009	0.020
Week 8	-3.5(-6.9 to -0.1)	0.742	-1.8(-5.2 to 1.7)	0.216	-3.4(-6.8 to 0.0)	0.031	0.122
Week 12	-3.6(-7.0 to -0.2)	0.735	-2.0 (-5.5 to 1.4)	0.224	-1.5(-4.9 to 1.8)	0.424	0.634
HOMA-IR							
Week 4	-1(-1.6 to -0.3)	0.051	-0.7(-1.4 to -0.1)	0.115	-1.2(-1.8 to -0.5)	<0.001	0.001
Week 8	-0.7(-1.4 to -0.1)	0.188	0.1(-0.6 to 0.7)	0.648	-0.8(-1.5 to -0.2)	0.003	0.003
Week 12	-0.6(-1.3 to 0.0)	0.249	-0.1(-0.7 to 0.6)	0.899	-0.5(-1.1 to 0.2)	0.018	0.041
Serum triglycerides, mg/dL							
Week 4	1.2(-32.5 to 34.9)	0.623	7.4(-26.8 to 41.6)	0.459	-35.8(-69.3 to -2.4)	0.003	<0.001
Week 8	-25.4(-59.1 to 8.2)	0.168	-14.1(-48.3 to 20.1)	0.514	-44.3(-77.8 to -10.9)	0.009	0.020
Week 12	-32.2(-65.9 to 1.4)	0.160	-2.2(-36.4 to 32.0)	0.862	-49.9(-83.3 to -16.4)	0.031	0.122
Serum total cholesterol, mg/dL						0.424	0.634
Week 4	-12.9(-47.4 to 21.5)	0.158	-0.7(-35.7 to 34.3)	0.522	-10.4(-44.6 to 23.8)		
Week 8	4.6(-29.9 to 39.1)	0.731	46.0 (11.0 to 80.9)	0.184	2.9(-31.4 to 37.1)	<0.0001	0.001
Week 12	1.7(-32.8 to 36.2)	0.102	1.2(-33.7 to 36.2)	0.850	-4.5(-38.8 to 29.7)	0.003	0.003
HDL-C, mg/dL						0.018	0.041
Week 4	0.3(-3.3 to 4.0)	0.214	0.4(-3.3 to 4.1)	0.738	-0.7(-4.3 to 2.9)		
Week 8	1.9(-1.7 to 5.5)	0.829	1.3(-2.4 to 5.0)	0.834	0.4(-3.2 to 4.0)	0.003	0.000
Week 12	6.6(2.9 to 10.2)	0.020	3.4(-0.3 to 7.1)	0.197	3.1(-0.6 to 6.7)	<0.0001	<0.0001
LDL-C, mg/dL						0.000	0.000
Week 4	-12.3(-22.7 to -2.0)	0.107	-5.4(-15.9 to 5.1)	0.021	-7.4(-17.7 to 2.9)		

Outcome	Between-group differences from baseline to week 12						*P* Value of Group differences
	LC diet vs NC diet		CR diet vs NC diet		LC+CR diet vs NC diet		
	Changes (95% CIs)	P Value	Changes (95% CIs)	P Value	Changes (95% CIs)	P Value	
Week 8	-8.8(-19.2 to 1.6)	0.580	-5.0 (-15.5 to 5.5)	0.056	-2.9(-13.1 to 7.4)	0.090	0.318
Week 12	-2.7(-13.0 to 7.7)	0.356	-3.2(-13.7 to 7.3)	0.140	-4.9(-15.2 to 5.4)	0.862	0.540
ALT, IU/L						0.719	0.154
Week 4	-7.4(-13.2 to -1.6)	0.168	-1.8(-7.7 to 4.1)	0.856	-2.2(-7.9 to 3.6)		
Week 8	-7.7(-13.5 to -1.9)	0.145	-3.1(-9.0 to 2.8)	0.465	-6.1(-11.9 to -0.3)	0.701	0.388
Week 12	-9.4(-15.2 to -3.6)	0.077	-2.9(-8.8 to 3.0)	0.593	-6.5(-12.3 to -0.7)	0.307	0.609
AST, IU/L						0.019	0.062
Week 4	-3.6(-6.8 to -0.4)	0.354	-2.4(-5.7 to 0.8)	0.654	-1.5(-4.6 to 1.7)		
Week 8	-2.1(-5.3 to 1.1)	0.877	-1.1(-4.4 to 2.1)	0.642	-1.7(-4.9 to 1.4)	0.243	0.132
Week 12	-5.6(-8.8 to -2.5)	0.044	-2.7(-6.0 to 0.5)	0.574	-3.8(-6.9 to -0.6)	0.935	0.156
Uric acid, mg/dl						0.683	0.109
Week 4	-0.6(-1.2 to 0.0)	0.167	-0.2(-0.8 to 0.4)	0.323	-0.2(-0.8 to 0.3)		
Week 8	-1.3(-1.8 to -0.7)	<0.0001	-0.5(-1.1 to 0.1)	0.013	-0.6(-1.2 to -0.1)	0.407	0.492
Week 12	-1.2(-1.8 to -0.6)	<0.0001	-0.6(-1.2 to -0.1)	0.003	-0.9(-1.4 to -0.3)	0.011	0.068
Visceral Fat Area, cm^2						0.593	0.098
Week 4	-5.3(-8.7 to -1.8)	0.003	-2.5(-6.0 to 1.1)	0.168	-8.6(-12.1 to -5.2)		
Week 8	-11.8(-15.4 to -8.2)	<0.0001	-5.5(-9.2 to -1.8)	0.004	-17.1(-20.7 to -13.5)	0.799	0.638
Week 12	-12.9(-17.2 to -8.7)	<0.0001	-5.2(-9.6 to -0.8)	0.020	-20.6(-24.8 to -16.3)	0.965	0.966
Body muscle rate, %						0.236	0.211
Week 4	-0.4(-2.2 to 1.4)	0.350	-0.5(-2.3 to 1.4)	0.289	0.0 (-1.8 to 1.8)		
Week 8	1.1(-0.7 to 2.9)	0.002	0.2(-1.6 to 2.1)	0.032	1.0 (-0.8 to 2.8)	0.294	0.551
Week 12	1.6(-0.2 to 3.4)	<0.0001	0.0 (-1.8 to 1.9)	0.000	1.9(0.1 to 3.6)	0.006	0.000

Table S8. Tests of between subject-effect

Source	P Value (Corrected Model)	P Value (Low-Carbohydrate Diet* Low-Calorie Diet)
BMI, kg/m^2	<0.001	<0.001
Weight, kg	0.008	<0.001
Waist circumference, cm	<0.001	<0.001
WHR ratio	0.015	<0.001
Body fat, %	<0.001	<0.001
Visceral Fat Area, cm^2	0.001	<0.001
Body muscle rate,%	0.002	<0.001
Plasma glucose, mg/dL	0.147	0.634
HOMA-IR	<0.001	0.041
Serum triglycerides, mg/dL	<0.001	<0.001
Serum total cholesterol, mg/dL	0.573	0.154
HDL-C, mg/dL	0.078	0.062
LDL-C, mg/dL	0.457	0.109
ALT, IU/L	<0.001	0.098
AST, IU/L	0.011	0.211
Uric acid, mg/dl	<0.001	<0.001
